# Novel phenoxy-((phenylethynyl) selanyl) propan-2-ol derivatives as potential anticancer agents

**DOI:** 10.1186/s13065-023-01076-0

**Published:** 2023-11-28

**Authors:** Wenxin Xu, Yali Du, Beibin Pan, Qiying Wang, Haoran Zheng, Ruonan Zhang, Jiaxin Lou, Guanghui Zhu, Jie Zhou, Jian Sun

**Affiliations:** 1 School of Pharmaceutical Sciences, Wenzhou Medical University, Zhejiang, China; 2 Sir Run Run Shaw Hospital, School of Medicine, Zhejiang University, Zhejiang, China; 3 The Second Affiliated Hospital, Yuying Children’s Hospital of Wenzhou Medical University, Zhejiang, China

**Keywords:** Selenocompounds, Synthesis, Structure-activity, Anticancer, Apoptosis

## Abstract

Selenocompounds protect against damage to healthy cells and induce the death of tumor cells by apoptosis; for this reason, they are attractive compounds for cancer research. In the present study, two series of novel phenoxy-((phenylethynyl) selanyl) propan-2-ol derivatives were synthesized, and their anti-proliferation activities were evaluated. Of the 23 compounds synthesized, most showed potent anti-proliferative activity against human cancer cell lines. Specifically, compounds **3h**, **3g**, and **3h-2**, which had a 2- or 4-position halogen substituent on 1-((phenylethynyl)selanyl)-3-phenoxypropan-2-ol, exhibited the best anti-proliferative activity against tumor cells. Flow cytometry demonstrated that **3h**, **3g**, and **3h-2** induced G2/M phase arrest and apoptosis in A549 cells. Cellular studies demonstrated that the induction of apoptosis by **3h** correlated with changes in the expression of cell cycle-related proteins and apoptosis-related proteins. Xenograft tumor experiments in nude mice revealed that compound **3h** has antitumor effects in vivo and no evident toxic effects in nude mice. In addition, compound **3h** alleviated cisplatin-induced liver and kidney damage. These findings uncover the applicability of compound **3h** as a novel lead compound for cancer treatment.

**Supplementary Information:**

The online version contains supplementary material available at 10.1186/s13065-023-01076-0.

## Introduction

There were almost 19.3 million new cancer cases worldwide in 2020 and more than 10.0 million deaths from cancer. By 2040, those figures will almost double [1]. The limitations of current cancer treatments include the lack of selectivity for healthy versus cancer cells, the development of multidrug resistance, and toxicities caused by most current cancer therapies. It is urgent to identify potential therapeutic targets and effective drugs [2, 3].

Apoptosis is a defense mechanism against the formation and progression of cancer, involving the activation, expression, and regulation of several genes [4]. Most current anticancer drugs work through the apoptotic signaling pathway to trigger cell death [5]. The selective regulation of tumor cell apoptosis has become the focus of antitumor drug research [6–8].

Selenium (Se) is a trace element with multiple roles in human health [9–11]. Selenoproteins mediate a series of biological effects, including antioxidant defense (protect normal cells from oxidative damage), anti-inflammatory actions, maintenance of thyroid function, and immune response regulation [12]. Epidemiologic evidence indicates an inverse association between Se status and cancer risk [13]. Some organoselenium compounds inhibit tumor cell growth in many xenograft models of cancer [14–16] and have synergistic effects when combined with chemotherapy drugs [17–20]. The effectiveness of selenium compounds as chemopreventive agents in vivo is associated with their ability to induce apoptosis, inhibit tumor cell migration and invasion, and regulate the cell cycle [21].

We have designed and synthesized 23 novel phenoxy-((phenylethynyl) selanyl) propan-2-ol derivatives. The proliferation inhibition, cell migration destruction, apoptosis, and cell cycle arrest in cancer cells were examined in relation to these compounds. Subsequently, the xenograft tumor in vivo experiment and protection of normal cells against cisplatin-induced damage were performed on the most potent derivative **3h**.

## Methods

### Reagents and antibodies

Fetal bovine serum (PA500) was purchased from Newzerum (Christchurch, New Zealand). DMEM- and RPMI 1640 medium Phosphate-buffered saline (PBS) and penicillin/streptomycin were purchased from Gibco. The DeadEnd™ Fluorometric TUNEL System TB235 kit (G3250) was from Promega (Southampton, UK), and the Annexin V-FITC/PI Apoptosis Detection Kit (A211-01, Vazyme) and MTT cell proliferation and cytotoxicity assay kit (G020-1-2, Nanjing Jiancheng) were also used. Paraformaldehyde was purchased from Macklin (Shanghai, China). Dimethyl sulfoxide (DMSO) was purchased from Solarbio (Beijing). DAPI was purchased from SouthernBiotech (Birmingham, USA). The following antibodies were used: Cyclin D1 (Abmart, T55404), Bcl2 (Abmart, T40056), Bax (Santa, SC-493), cleaved Caspase 3 (Affinity Biosciences, AF7022) and β-actin (TransGen Biotech, HC201-01).

### Synthesis procedures of 1-phenoxy-3-((phenylethynyl) selanyl) propan-2-ol derivatives

The synthesis procedure of 1-phenoxy-3-((phenylethynyl) selanyl) propan-2-ol derivatives was a copper-catalyzed three-component cascade, as previously reported [22]. Briefly, propionic acids (0.2 mmol), epoxides (0.6 mmol), Se (0.6 mmol), CuCl_2_ (0.02 mmol), TBAI (0.4 mmol), 1, 10-phen (0.02 mmol), and Cs_2_CO_3_ (0.6 mmol) in H_2_O (2 mL) were placed in a 25-mL glass. The reaction mixture was stirred at 50 ℃ for 24 h. After it was cooled, 10 mL of ethyl acetate was added, and the mixture was filtered through diatomaceous earth. The organic phase was dried over Na_2_SO_4_ and concentrated under reduced pressure. The residue was then purified by chromatography to provide the final product.

Characterizations of products in detail are as follows:

#### 1-phenoxy-3-(phenylethynylselanyl) propan-2-ol (3a)

Following the general procedure, using (petroleum ether: EtOAc = 7: 1) as the eluant to afford yellow oil liquid (60.4 mg, 91% yield). ^**1**^** H NMR** (500 MHz, CDCl_3_): δ 7.36–7.34 (m, 2 H), 7.27–7.23 (m, 5 H), 6.97–6.90 (m, 3 H), 4.37 (brs, 1 H), 4.16–4.10 (m, 2 H), 3.17 (dd, *J* = 12.50, 5.40 Hz, 1 H), 3.07 (dd, *J* = 12.50, 6.90 Hz, 1 H), 2.84–2.83 (m, 1 H); ^**13**^** C NMR** (125 MHz, CDCl_3_): δ 158.4, 131.6, 129.6, 128.4, 128.3, 123.2, 121.4, 114.7, 99.6, 70.3, 69.6, 69.5, 32.8.

#### 1-phenoxy-3-(p-tolylethynylselanyl)propan-2-ol (3b)

Following the general procedure, using (petroleum ether: EtOAc = 7: 1) as the eluant to afford light yellow solid (65.7 mg, 95% yield), Mp = 49–50 ℃. ^**1**^** H NMR** (500 MHz, CDCl_3_): δ 7.29–7.25 (m, 4 H), 7.08 (d, *J* = 7.90 Hz, 2 H), 6.96 (t, *J* = 7.40 Hz, 1 H), 6.92 (d, *J* = 8.20 Hz, 2 H), 4.39–4.37 (m, 1 H), 4.18–4.12 (m, 2 H), 3.17 (dd, *J* = 12.40, 5.25 Hz, 1 H), 3.07 (dd, *J* = 12.40, 5.25 Hz, 1 H), 2.77–2.76 (m, 1 H), 2.33 (s, 3 H); ^**13**^** C NMR** (125 MHz, CDCl_3_): δ 158.3, 138.6, 131.6, 129.6, 129.1, 121.3, 120.1, 114.6, 99.8, 70.3, 69.6, 68.3, 32.7, 21.5; **HRMS** (ESI): calcd for C_18_H_18_O_2_Se [M + H]^+^ 347.0544, found 347.0540.

#### 1-(mesitylethynylselanyl)-3-phenoxypropan-2-ol (3c)

Following the general procedure, using (petroleum ether: EtOAc = 7: 1) as the eluant to afford yellow oil liquid (58.3 mg, 78% yield). ^**1**^** H NMR** (500 MHz, CDCl_3_): δ 7.27–7.23 (m, 2 H), 6.95 (t, *J* = 7.30 Hz, 1 H), 6.90 (d, *J* = 8.15 Hz, 1 H), 6.83 (s, 2 H), 4.41–4.38 (m, 1 H), 4.17–4.11 (m, 2 H), 3.19 (dd, *J* = 12.50, 5.40 Hz, 1 H), 3.06 (dd, *J* = 12.50, 5.40 Hz, 1 H), 2.76–2.75 (m, 1 H), 2.36 (s, 6 H), 2.26 (s, 3 H); ^**13**^** C NMR** (125 MHz, CDCl_3_): δ 158.4, 140.3, 137.8, 129.5, 127.6, 121.3, 120.1, 114.6, 97.7, 75.7, 70.4, 69.6, 33.3, 21.2, 20.9; **HRMS** (ESI): calcd for C_20_H_22_O_2_Se [M + H]^+^ 375.0858, found 375.0853.

#### 1-((4-tert-butylphenyl)ethynylselanyl)-3-phenoxypropan-2-ol (3d)

Following the general procedure, using (petroleum ether: EtOAc = 7: 1) as the eluant to afford yellow oil liquid (72.9 mg, 94% yield). ^**1**^** H NMR** (500 MHz, CDCl_3_): δ 7.31–7.27 (m, 6 H), 6.98–6.91 (m, 3 H), 4.39 (dt, *J* = 16.50, 5.30 Hz, 1 H), 4.19–4.13 (m, 2 H), 3.18 (dd, *J* = 12.50, 5.40 Hz, 1 H), 3.08 (dd, *J* = 12.50, 5.40 Hz, 1 H), 1.30 (s, 9 H); ^**13**^** C NMR** (125 MHz, CDCl_3_): δ 158.3, 151.8, 131.4, 129.5, 125.3, 131.3, 120.1, 114.6, 99.7, 70.3, 69.6, 68.3, 34.8, 32.8, 31.1; **HRMS** (ESI): calcd for C_21_H_24_O_2_Se [M + H]^+^ 389.1014, found 389.1007.

#### 1-(biphenyl-4-ylethynylselanyl)-3-phenoxypropan-2-ol (3e)

Following the general procedure, using (petroleum ether: EtOAc = 7: 1) as the eluant to afford light yellow solid (59.6 mg, 73% yield), Mp = 78–79 ℃. ^**1**^** H NMR** (500 MHz, CDCl_3_): δ 7.59–7.56 (m, 2 H), 7.51–7.50 (m, 2 H), 7.44–7.41 (m, 4 H), 7.36–7.33 (m, 1 H), 7.28–7.24 (m, 2 H), 6.98–6.92 (m, 3 H), 4.39 (brs, 1 H), 4.19–4.14 (m, 2 H), 3.21 (dd, *J* = 12.50, 5.40 Hz, 1 H), 3.10 (dd, *J* = 12.50, 5.40 Hz, 1 H), 2.77 (brs, 1 H); ^**13**^** C NMR** (125 MHz, CDCl_3_): δ 158.3, 141.1, 140.3, 132.9, 132.0, 129.6, 128.9, 127.7, 127.1, 127.0, 127.0, 126.9, 122.0, 121.4, 114.6, 99.5, 70.3, 70.2, 69.6, 32.9; **HRMS** (ESI): calcd for C_23_H_20_O_2_Se [M + H]^+^ 409.0701, found 409.0732.

#### 1-((4-methoxyphenyl)ethynylselanyl)-3-phenoxypropan-2-ol (3f)

Following the general procedure, using (petroleum ether: EtOAc = 7: 1) as the eluant to afford light yellow solid (65.2 mg, 90% yield), Mp = 59–60 ℃. ^**1**^** H NMR** (500 MHz, CDCl_3_): δ 7.33–7.27 (m, 4 H), 6.97 (t, *J* = 7.3 Hz, 1 H), 6.93–6.91 (m, 2 H), 6.82–6.80 (m, 2 H), 4.39 (brs, 1 H), 4.19–4.13 (m, 2 H), 3.80 (s, 3 H), 3.17 (dd, *J* = 12.50, 5.40 Hz, 1 H), 3.07 (dd, *J* = 12.50, 5.40 Hz, 1 H), 2.74 (brs, 1 H); ^**13**^** C NMR** (125 MHz, CDCl_3_): δ 159.8, 158.3, 133.4, 129.5, 121.3, 115.3, 114.6, 113.9, 99.5, 70.3, 69.6, 67.3, 55.3, 32.8; **HRMS** (ESI): calcd for C_18_H_18_O_3_Se [M + H]^+^ 363.0494.

#### 1-((4-fluorophenyl)ethynylselanyl)-3-phenoxypropan-2-ol (3g)

Following the general procedure, using (petroleum ether: EtOAc = 7: 1) as the eluant to afford yellow oil liquid (43.4 mg, 62% yield). ^**1**^** H NMR** (500 MHz, CDCl_3_): δ 7.32–7.24 (m, 4 H), 6.97–6.89 (m, 5 H), 4.37–4.34 (m, 1 H), 4.15–4.10 (m, 2 H), 3.17 (dd, *J* = 12.50, 5.50 Hz, 1 H), 3.07 (dd, *J* = 12.51, 5.50 Hz, 1 H), 2.85 (d, *J* = 5.40 Hz, 1 H); ^**13**^** C NMR** (125 MHz, CDCl_3_): δ 162.5 (d, *J*_F_ = 249.9 Hz), 158.4 (d, *J*_F_ = 19.7 Hz), 133.6 (d, *J*_F_ = 8.3 Hz), 129.6, 121.4, 121.3, 119.3 (d, *J*_F_ = 3.2 Hz), 115.6, 115.5, 114.7, 98.4, 70.3, 69.5, 69.4, 32.8; ^**19**^** F NMR** (470 MHz, CDCl_3_): δ -110.37 (s, 1 F); **HRMS** (ESI): calcd for C_17_H_15_FO_2_Se [M + H]^+^ 351.0294, found 351.0283.

#### 1-((2-chlorophenyl)ethynylselanyl)-3-phenoxypropan-2-ol (3h)

Following the general procedure, using (petroleum ether: EtOAc = 7: 1) as the eluant to afford yellow oil liquid (60.0 mg, 82% yield). ^**1**^** H NMR** (500 MHz, CDCl_3_): δ 7.34–7.32 (m, 2 H), 7.27–7.22 (m, 2 H), 7.18–7.12 (m, 2 H), 6.94–6.88 (m, 3 H), 4.41–4.40 (m, 1 H), 4.12–4.11 (m, 2 H), 3.20 (dd, *J* = 12.50, 5.40 Hz, 1 H), 3.09 (dd, *J* = 12.50, 5.40 Hz, 1 H), 2.94–2.93 (d, *J* = 3.90 Hz, 1 H); ^**13**^** C NMR** (125 MHz, CDCl_3_): δ 158.4, 135.6, 133.0, 129.6, 129.2, 129.1, 126.5, 123.2, 121.4, 121.3, 114.7, 96.5, 76.1, 70.4, 69.5, 33.2; **HRMS** (ESI): calcd for C_17_H_15_ClO_2_Se [M + H]^+^ 366.9998, found 366.9999.

#### 1-((3-chlorophenyl)ethynylselanyl)-3-phenoxypropan-2-ol (3i)

Following the general procedure, using (petroleum ether: EtOAc = 7: 1) as the eluant to afford yellow oil liquid (62.2 mg, 85% yield). ^**1**^** H NMR** (500 MHz, CDCl_3_): δ 7.30–7.29 (m, 1 H), 7.27–7.22 (m, 3 H), 7.19–7.14 (m, 2 H), 6.97–6.94 (m, 1 H), 6.90–6.88 (m, 2 H), 4.36–4.33 (m, 1 H), 4.15–4.09 (m, 2 H), 3.18 (dd, *J* = 12.45, 5.50 Hz, 1 H), 3.07 (dd, *J* = 12.45, 5.50 Hz, 1 H), 2.91–2.90 (m, 1 H); ^**13**^** C NMR** (125 MHz, CDCl_3_): δ 158.3, 134.1, 131.3, 129.6, 129.5, 128.6, 124.8, 121.5, 114.7, 114.6, 98.2, 71.8, 70.2, 69.5, 32.9; **HRMS** (ESI): calcd for C_17_H_15_ClO_2_Se [M + H]^+^ 366.9998, found 366.9999.

#### 1-((4-bromophenyl)ethynylselanyl)-3-phenoxypropan-2-ol (3j)

Following the general procedure, using (petroleum ether: EtOAc = 7: 1) as the eluant to afford light yellow solid (65.6 mg, 80% yield), Mp = 74–75 ℃. ^**1**^** H NMR** (500 MHz, CDCl_3_): δ 7.38–7.36 (m, 2 H), 7.27–7.23 (m, 2 H), 7.16–7.14 (m, 2 H), 6.97–6.93 (m, 1 H), 6.90–6.88 (m, 2 H), 4.36–4.33 (m, 1 H), 4.15–4.09 (m, 2 H), 3.18 (dd, *J* = 12.43, 5.50 Hz, 1 H), 3.07 (dd, *J* = 12.43, 5.50 Hz, 1 H), 2.88–2.83 (m, 1 H); ^**13**^** C NMR** (125 MHz, CDCl_3_): δ 158.3, 132.9, 131.5, 129.6, 129.5, 122.5, 122.1, 121.4, 121.3, 114.6, 98.5, 71.2, 70.2, 69.5, 32.8; **HRMS** (ESI): calcd for C_17_H_15_BrO_2_Se [M + H]^+^ 410.9493, found 410.9504.

#### 4-((2-hydroxy-3-phenoxypropylselanyl)ethynyl)benzonitrile (3k)

Following the general procedure, using (petroleum ether: EtOAc = 7: 1) as the eluant to afford light yellow solid (46.4 mg, 65% yield), Mp = 71–72 ℃. ^**1**^** H NMR** (500 MHz, CDCl_3_): δ 7.55 (d, *J* = 8.1 Hz, 2 H), 7.35 (d, *J* = 8.1 Hz, 2 H), 7.30 (t, *J* = 7.8 Hz, 2 H), 7.00 (t, *J* = 7.4 Hz, 1 H), 6.94 (d, *J* = 8.1 Hz, 2 H), 4.42–4.37 (m, 1 H), 4.18 (d, *J* = 4.9 Hz, 2 H), 3.28 (dd, *J* = 12.5, 5.6 Hz, 1 H), 3.17 (dd, *J* = 12.5, 6.6 Hz, 1 H), 2.80 (brs, 1 H); ^**13**^** C NMR** (125 MHz, CDCl_3_): δ 158.2, 131.9, 131.6, 129.6, 127.9, 131.5, 118.4, 114.6, 111.2, 98.2, 76.3, 69.9, 69.4, 33.1; **HRMS** (ESI): calcd for C_18_H_15_NO_2_Se [M + H]^+^ 358.0340, found 358.0325.

#### 1-phenoxy-3-(thiophen-3-ylethynylselanyl)propan-2-ol (3L)

Following the general procedure, using (petroleum ether: EtOAc = 7: 1) as the eluant to afford yellow oil liquid (54.7 mg, 81% yield). ^**1**^** H NMR** (500 MHz, CDCl_3_): δ 7.33–7.27 (m, 3 H), 7.21 (dd, J = 3.6, 1.2 Hz, 1 H), 7.02–6.95 (m, 4 H), 4.42–4.38 (m, 1 H), 4.21–4.14 (m, 2 H), 3.21 (dd, J = 12.5, 5.4 Hz, 1 H), 3.11 (dd, J = 12.4, 6.9 Hz, 1 H), 2.79 (brs, 1 H);^**13**^** C NMR** (125 MHz, CDCl_3_): δ158.4, 129.9, 129.6, 129.5, 125.2, 122.3, 121.4, 121.3, 114.7, 94.5, 70.3, 69.5, 69.1, 68.8, 32.8; **HRMS** (ESI): calcd for C_15_H_14_O_2_SSe [M + H]^+^ 338.9952, found 338.9954.

#### 1-(phenylethynylselanyl)-3-(p-tolyloxy)propan-2-ol (4a)

Following the general procedure, using (petroleum ether: EtOAc = 7: 1) as the eluant to afford yellow oil liquid (63.6 mg, 92% yield). ^**1**^** H NMR** (500 MHz, CDCl_3_): δ 7.36–7.34 (m, 2 H), 7.30–7.24 (m, 3 H), 7.05 (d, *J* = 8.40 Hz, 2 H), 6.81 (d, *J* = 8.40 Hz, 2 H), 4.37–4.35 (m, 1 H), 4.15–4.09 (m, 2 H), 3.18 (dd, *J* = 12.50, 5.40 Hz, 1 H), 3.08 (dd, *J* = 12.5, 5.40 Hz, 1 H), 2.76 (brs, 1 H), 2.27 (s, 3 H); ^**13**^** C NMR** (125 MHz, CDCl_3_): δ 156.2, 131.6, 130.6, 130.0, 128.3, 128.2, 123.2, 114.5, 99.6, 70.5, 69.6, 69.5, 32.8, 20.4; **HRMS** (ESI): calcd for C_18_H_18_O_2_Se [M + H]^+^ 347.0545, found 347.0541.

#### 1-(4-bromophenoxy)-3-(phenylethynylselanyl)propan-2-ol (4b)

Following the general procedure, using (petroleum ether: EtOAc = 7: 1) as the eluant to afford yellow oil liquid (61.5 mg, 75% yield). ^**1**^** H NMR** (500 MHz, CDCl_3_): δ 7.35–7.25 (m, 7 H), 6.81–6.77 (m, 2 H), 4.38–4.36 (s, 1 H), 4.15–4.09 (m, 2 H), 3.17 (dd, *J* = 12.50, 5.40 Hz, 1 H), 3.07 (dd, *J* = 12.50, 5.40 Hz, 1 H), 2.74 (brs, 1 H); ^**13**^** C NMR** (125 MHz, CDCl_3_): δ 157.4, 132.4, 131.6, 128.4, 128.3, 123.0, 116.4, 113.5, 99.7, 70.5, 69.4, 69.2, 32.7.

#### 1-(phenylethynylselanyl)-3-(4-(trifluoromethoxy)phenoxy)propan-2-ol (4c)

Following the general procedure, using (petroleum ether: EtOAc = 7: 1) as the eluant to afford light yellow solid (64.1 mg, 77% yield), Mp = 48–49 ℃. ^**1**^** H NMR** (500 MHz, CDCl_3_): δ 7.35–7.34 (m, 5 H), 7.12–7.11 (m, 2 H), 6.91–6.89 (m, 2 H), 4.39–4.38 (m, 1 H), 4.18–4.12 (m, 2 H), 3.18 (dd, *J* = 12.50, 5.40 Hz, 1 H), 3.08 (dd, *J* = 12.50, 5.40 Hz, 1 H), 2.74–2.73 (m, 1 H); ^**13**^** C NMR** (125 MHz, CDCl_3_): δ 156.8, 143.2, 131.5, 128.4, 128.3, 123.6, 123.0, 122.4, 121.5, 119.5, 117.5, 115.4, 99.7, 70.7, 69.4, 69.2, 32.7; ^**19**^** F NMR** (470 MHz, CDCl_3_): δ -58.4 (s, 3 F). **HRMS** (ESI): calcd for C_18_H_15_F_3_O_3_Se [M + H]^+^ 417.0211, found 417.0216.

#### 1-(4-nitrophenoxy)-3-(phenylethynylselanyl)propan-2-ol (4d)

Following the general procedure, using (petroleum ether: EtOAc = 7: 1) as the eluant to afford light yellow solid (54.3 mg, 72% yield), Mp = 71–72 ℃. ^**1**^** H NMR** (500 MHz, CDCl_3_): δ 8.16–8.12 (m, 2 H), 7.33–7.24 (m, 5 H), 6.99–6.96 (m, 2 H), 4.45–4.42 (m, 1 H), 4.30–4.25 (m, 2 H), 3.20 (dd, *J* = 12.50, 5.40 Hz, 4 H), 3.09 (dd, *J* = 12.50, 5.40 Hz, 1 H), 2.73–2.72 (m, 1 H); ^**13**^** C NMR** (125 MHz, CDCl_3_): δ 163.2, 141.9, 131.6, 128.6, 128.3, 125.9, 122.8, 114.6, 99.8, 70.8, 69.3, 68.9, 32.5.

#### 1-(3-(diethylamino)phenoxy)-3-(phenylethynylselanyl)propan-2-ol (4e)

Following the general procedure, using (petroleum ether: EtOAc = 7: 1) as the eluant to afford yellow oil liquid (66.9 mg, 83% yield). ^**1**^** H NMR** (500 MHz, CDCl_3_): δ 7.38–7.36 (m, 2 H), 7.28–7.25 (m, 3 H), 7.10–7.07 (m, 1 H), 6.33–6.31 (m, 1 H), 6.24–6.21 (m, 2 H), 4.37–4.36 (m, 1 H), 4.17–4.11(m, 2 H), 3.30 (dd, *J* = 14.0, 7.0 Hz, 4 H), 3.19 (dd, *J* = 12.50, 5.40 Hz, 1 H), 3.09 (dd, *J* = 12.50, 5.40 Hz, 1 H), 2.74–2.73 (m, 1 H), 1.14 (s, 6 H); ^**13**^** C NMR** (125 MHz, CDCl_3_): δ 159.7, 149.3, 131.6, 130.0, 128.3, 123.2, 105.7, 100.8, 99.6, 98.9, 70.1, 69.6, 69.5, 44.4, 32.9, 12.6; **HRMS** (ESI): calcd for C_21_H_25_NO_2_Se [M + H]^+^ 404.1123, found 404.1129.

#### 1-(naphthalen-2-yloxy)-3-(phenylethynylselanyl)propan-2-ol (4f)

Following the general procedure, using (petroleum ether: EtOAc = 7: 1) as the eluant to afford light yellow solid (68.7 mg, 90% yield), Mp = 63–64 ℃. ^**1**^** H NMR** (500 MHz, CDCl_3_): δ 7.76–7.71 (m, 2 H), 7.65 (d, *J* = 8.18 Hz, 1 H), 7.43–7.40 (m, 1 H), 7.35–7.32 (m, 3 H), 7.28–7.20 (m, 3 H), 7.17–7.15 (m, 2 H), 4.48–4.43 (m, 1 H), 4.31–4.25 (m, 2 H), 3.23 (dd, *J* = 12.50, 5.40 Hz, 4 H), 3.13 (dd, *J* = 12.50, 5.40 Hz, 1 H), 2.81 (brs, 1 H); ^**13**^** C NMR** (125 MHz, CDCl_3_): δ 156.3, 134.4, 131.6, 129.6, 129.3, 128.3, 128.2, 127.6, 126.8, 126.5, 123.9, 123.1, 118.6, 107.1, 99.7, 70.4, 69.6, 69.4, 32.8; **HRMS** (ESI): calcd for C_21_H_18_O_2_Se [M + H]^+^ 383.0544, found 383.0537.

#### 1-(benzo[d][1,3]dioxol-5-yloxy)-3-(phenylethynylselanyl)propan-2-ol (4g)

Following the general procedure, using (petroleum ether: EtOAc = 7 : 1) as the eluant to afford yellow oil liquid (58.6 mg, 78% yield). ^**1**^** H NMR** (500 MHz, CDCl_3_): δ 7.37–7.35 (m, 2 H), 7.29–7.27 (m, 3 H), 6.67 (d, *J* = 8.45 Hz, 1 H), 6.50 (d, *J* = 2.45 Hz, 1 H), 6.34 (dd, *J* = 8.45 Hz, 2.45 Hz, 1 H), 5.90 (s, 2 H), 4.36–4.33 (m, 1 H), 4.11–4.05 (m, 2 H), 3.17 (dd, *J* = 12.50, 5.40 Hz, 4 H), 3.07 (dd, *J* = 12.50, 5.40 Hz, 1 H), 2.72 (d, *J* = 5.15 Hz, 1 H); ^**13**^** C NMR** (125 MHz, CDCl_3_): δ 153.8, 148.3, 142.1, 131.6, 128.3, 128.2, 123.1, 107.9, 105.9, 101.2, 99.6, 98.3, 71.3, 69.5, 69.4, 32.8; **HRMS** (ESI): calcd for C_18_H_16_O_4_Se [M + H]^+^ 377.0286, found 377.0280.

#### 1-(5-isopropyl-2-methylphenoxy)-3-(phenylethynylselanyl)propan-2-ol (4h)

Following the general procedure, using (petroleum ether: EtOAc = 7: 1) as the eluant to afford yellow oil liquid (60.5 mg, 78% yield). ^**1**^** H NMR** (500 MHz, CDCl_3_): δ 7.34–7.32 (m, 2 H), 7.28–7.24 (m, 3 H), 7.04 (d, *J* = 7.60 Hz, 1 H), 6.76–6.74 (m, 1 H), 6.70 (s, 1 H), 4.42–4.39 (m, 1 H), 4.18 (d, *J* = 4.95 Hz, 2 H), 3.23 (dd, *J* = 14.0, 7.0 Hz, 4 H), 3.11 (dd, *J* = 12.4, 6.3 Hz, 1 H), 2.84–2.78 (m, 1 H), 2.19 (s, 3 H), 1.20 (d, *J* = 6.90 Hz, 6 H); ^**13**^** C NMR** (125 MHz, CDCl_3_): δ 156.3, 148.1, 131.5, 130.6, 128.3, 124.0, 123.2, 118.8, 109.8, 99.5, 70.3, 69.8, 69.5, 34.1, 33.0, 24.1, 15.8; **HRMS** (ESI): calcd for C_21_H_24_O_2_Se [M + H]^+^ 389.1014, found 389.1010.

#### 1-(((2-fluorophenyl)ethynyl)selanyl)-3-phenoxypropan-2-ol (3 h-1)

Following the general procedure, using (petroleum ether: EtOAc = 9:1) as the eluent afforded a yellow liquid (53.9 mg, 77% yield). ^**1**^** H NMR** (400 MHz, CDCl_3_): δ 7.38 (td, *J* = 7.6, 1.9 Hz, 1 H), 7.31 (dd, *J* = 10.7, 5.2 Hz, 3 H), 7.10 (td, *J* = 8.7, 8.0, 2.9 Hz, 2 H), 6.99 (dd, *J* = 18.0, 7.8 Hz, 3 H), 4.45 (q, *J* = 5.5 Hz, 1 H), 4.24–4.17 (m, 2 H), 3.26 (dd, *J* = 12.5, 5.4 Hz, 1 H), 3.16 (dd, *J* = 12.5, 6.9 Hz, 1 H), 2.97 (t, *J* = 6.6 Hz, 1 H); ^**13**^** C NMR** (100 MHz, CDCl_3_): δ 162.91 (d, *J* = 251.3 Hz), 158.39, 133.43, 130.08 (d, *J* = 7.9 Hz), 129.66, 124.04 (d, *J* = 3.7 Hz), 121.41, 115.56 (d, *J* = 20.8 Hz), 114.68, 111.90 (d, *J* = 15.7 Hz), 92.90, 75.62, 70.36, 69.56, 33.04; ^**19**^** F NMR** (375 MHz, CDCl_3_): -109.96; **HRMS** (ESI): calcd for C_17_H_16_FO_2_Se [M + H]^+^ 351.0299, found 351.0301.

#### 1-(((2-bromophenyl)ethynyl)selanyl)-3-phenoxypropan-2-ol (3h-2)

Following the general procedure, using (petroleum ether: EtOAc = 9:1) as the eluant afforded a yellow liquid (64.8 mg, 79% yield). ^**1**^** H NMR** (400 MHz, CDCl_3_): δ 7.60 (dd, *J* = 8.0, 1.3 Hz, 1 H), 7.42 (dd, *J* = 7.7, 1.7 Hz, 1 H), 7.35–7.30 (m, 2 H), 7.26 (td, *J* = 7.6, 1.3 Hz, 1 H), 7.17 (td, *J* = 7.7, 1.7 Hz, 1 H), 7.03–6.97 (m, 3 H), 4.51 (q, *J* = 5.4 Hz, 1 H), 4.24–4.17 (m, 2 H), 3.30 (dd, *J* = 12.4, 5.3 Hz, 1 H), 3.17 (dd, *J* = 12.4, 7.0 Hz, 1 H), 3.03 (t, *J* = 5.6 Hz, 1 H); ^**13**^** C NMR** (100 MHz, CDCl_3_): δ 158.39, 133.15, 132.44, 129.69, 129.39, 127.16, 125.37, 125.24, 121.43, 114.72, 98.34, 75.69, 70.41, 69.53, 33.17; **HRMS** (ESI): calcd for C_17_H_16_BrO_2_Se [M + H]^+^ 410.9499, found 410.9502.

#### 1-(((2-methoxyphenyl)ethynyl)selanyl)-3-phenoxypropan-2-ol (3h-3)

Following the general procedure, using (petroleum ether: EtOAc = 9:1) as the eluent afforded a yellow liquid (65.9 mg, 91% yield). ^**1**^** H NMR** (400 MHz, CDCl_3_): δ 7.38 (dd, *J* = 7.6, 1.8 Hz, 1 H), 7.34–7.30 (m, 3 H), 7.00 (dd, *J* = 17.5, 7.9 Hz, 3 H), 6.95–6.88 (m, 2 H), 4.51 (q, *J* = 5.5 Hz, 1 H), 4.24–4.17 (m, 2 H), 3.88 (d, *J* = 1.3 Hz, 3 H), 3.27–3.11 (m, 3 H); ^**13**^** C NMR** (100 MHz, CDCl_3_): δ 160.24, 158.49, 133.31, 129.88, 129.64, 121.32, 120.54, 114.68, 112.45, 110.62, 96.17, 73.59, 70.54, 69.86, 55.80, 32.89; **HRMS** (ESI): calcd for C_18_H_19_O_3_Se [M + H]^+^ 363.0499, found 363.0500.

### Cell culture

The human lung adenocarcinoma cell line A549, the human hepatoblastoma cell line HepG2, and the human colon carcinoma cell line RKO were purchased from the Shanghai Cell Bank of the Chinese Academy of Sciences (Shanghai, China). These cells were cultured in RPMI 1640 or DMEM (4.5 g/l glucose) supplemented with 10% fetal bovine serum in a humidified incubator with 5% CO_2_ at 37 °C.

### Cell viability assay

Compounds were dissolved in DMSO and diluted with the culture medium. For the MTT assay, the cells were grown in 96-well plates (5 × 10^3^ cells/well) for 24 h and treated with various concentrations of synthetic compounds for 48 h. The cells were incubated with the MTT assay kit for another 4 h. The dark blue crystals (formazan) were dissolved in DMSO. The absorbance was measured using a multifunction microplate reader (Molecular Devices, Flex Station 3) at 570 nm. The cytotoxic effects of each compound were expressed as IC50 values. All experiments were performed in triplicate in three independent experiments.

### Flow cytometric analysis

A549 cells were incubated in six-well plates (3 × 10^5^ cells/well) for 24 h. The cells were treated in the presence or absence of compounds **3h**, **3g**, and **3h-2** at 1 µmol for 12 h. The cells were then incubated with an Annexin V-FITC/PI Apoptosis Detection Kit according to the manufacturer’s instructions. Almost 10,000 events were collected for each sample and were analyzed by flow cytometry (Beckman Coulter, Epics XL). The results were calculated using EXPO32 ADC analysis software.

### Western blot analysis

The harvested cells were lysed with cold RIPA lysis buffer (Solarbio, Beijing, China) with a protease inhibitor cocktail (Thermo Fisher Scientific, MA) to obtain total protein. Protein concentrations were determined using the modified Coomassie bright blue method. An aliquot of protein was separated using different sodium dodecyl sulfate‒polyacrylamide gel electrophoresis and transferred onto 0.45-µm polyvinylidene difluoride membranes (Solarbio, Beijing, China). Blots were cut prior to hybridization with antibodies during blotting. Membranes were blocked with 5% skim milk at room temperature for 2 h and incubated overnight at 4 °C with primary antibodies against Cyclin D1 (1:1000), Bax (1:1000), Bcl2 (1:1000), cleaved caspase 3 (1:1000), or β-actin (1:1000). Protein bands were visualized using a chemiluminescence reagent (TransGen Biotech, DW101-01) with horseradish peroxidase-conjugated secondary antibodies and quantified using ImageJ software (version 1.44p, NIH).

### TUNEL assay

A549 cells were fixed with 4% paraformaldehyde and stained using a TUNEL System TB235 kit. Stained cells were visualized using a Nikon confocal microscope.

### Cell migration assay

A549 cells were seeded in 24-well plates (1 × 10^4^ per well) and grown to confluence before serum starvation for 12 h. The monolayers were scratched with a 10-µl pipet or needle tip. We removed the culture medium and washed the cells with PBS three times to eliminate floating cells. The medium for cell culture was replaced with serum-free RPMI 1640 medium and incubated with compounds **3h**, **3g**, and **3h-2 (**1 µmol). The changes in the wound area were measured 24 or 48 h later. The cell images were detected using a light microscope (Nikon, Japan).

### RNA isolation and real-time PCR

Total RNA was extracted from A549 cells incubated for 24 h with or without the addition of compounds of 1 µmol **3h** using Trizol reagent. We used a NanoDrop™ One device (Thermo Scientific, ND-ONE-W) to measure RNA concentrations. cDNA was reverse-transcribed using a SuperScript™ II Reverse Transcriptase kit (TransGen Biotech, AT341). qRT‒PCR was performed using PerfectStart™ Green qPCR SuperMix (TransGen Biotech, AQ601) on a CFX96 Touch Real-Time PCR Detection System (Bio-Rad, Inc., Hercules, USA). The primers are listed in Supplementary Table S1. The relative amounts of the mRNAs were expressed as 2^−ΔΔCT^.

### In vivo xenograft Tumor model study

Five-week-old male BALB/c nude mice (18–20 g, *n* = 10) were purchased from Charles River Laboratories (Beijing, China). The Animal Care and Use Committee of Wenzhou Medical University, China, approved all animal procedures. The nude mice were maintained in an animal chamber free of pathogens and fed with sterilized water and chow. The xenograft model was established by subcutaneous injection of A549 cells in the logarithmic growth phase into the right axilla. When the tumor volume reached approximately 100 mm^3^, the xenograft tumor-bearing nude mice were randomly divided into two groups (five in each setting): vehicle-treated and **3h**-treated. The mice in the **3h**-treated group were intraperitoneally injected with compound **3h** (2.5 mg/kg, once a day), and the mice in the vehicle-treated group were intraperitoneally injected with the same volume of PBS. The body weight and tumor volume were measured every other day during the experiment. The tumor volume was calculated as follows: volume (mm^3^) = 0.5 × length (mm) × width (mm)^2^. After two weeks, all mice were euthanized with pentobarbital sodium (100 mg/kg, i.p.), and the tumors were peeled off, photographed, harvested, and weighed.

### Protection of Selenocompounds against cisplatin damage to normal cells

Prior to treatment, renal podocytes (MPC) and liver (AML-12) cells were cultured in serum-free media for 10–12 h and divided into: (i) Normal group; (ii) Cisplatin group (20 µmol cisplatin), and (iii) Compound **3h** intervention groups (20 µmol cisplatin-treated with 0.01, 0.1, 1, 10 µmol compounds **3h**). MTT and TUNEL experiments were carried out.

### Statistical analysis

All statistical analyses were performed using GraphPad Prism 9.0. Data are presented as the mean ± SEM. Each set of experiments was repeated independently at least three times. Student’s-t test was performed to determine the significance of differences between pairs. For the comparison of more than two groups, one-way ANOVA was used. A value of P < 0.05 was considered statistically significant.

## Results and discussion

### Chemistry

**Fig. 1:**
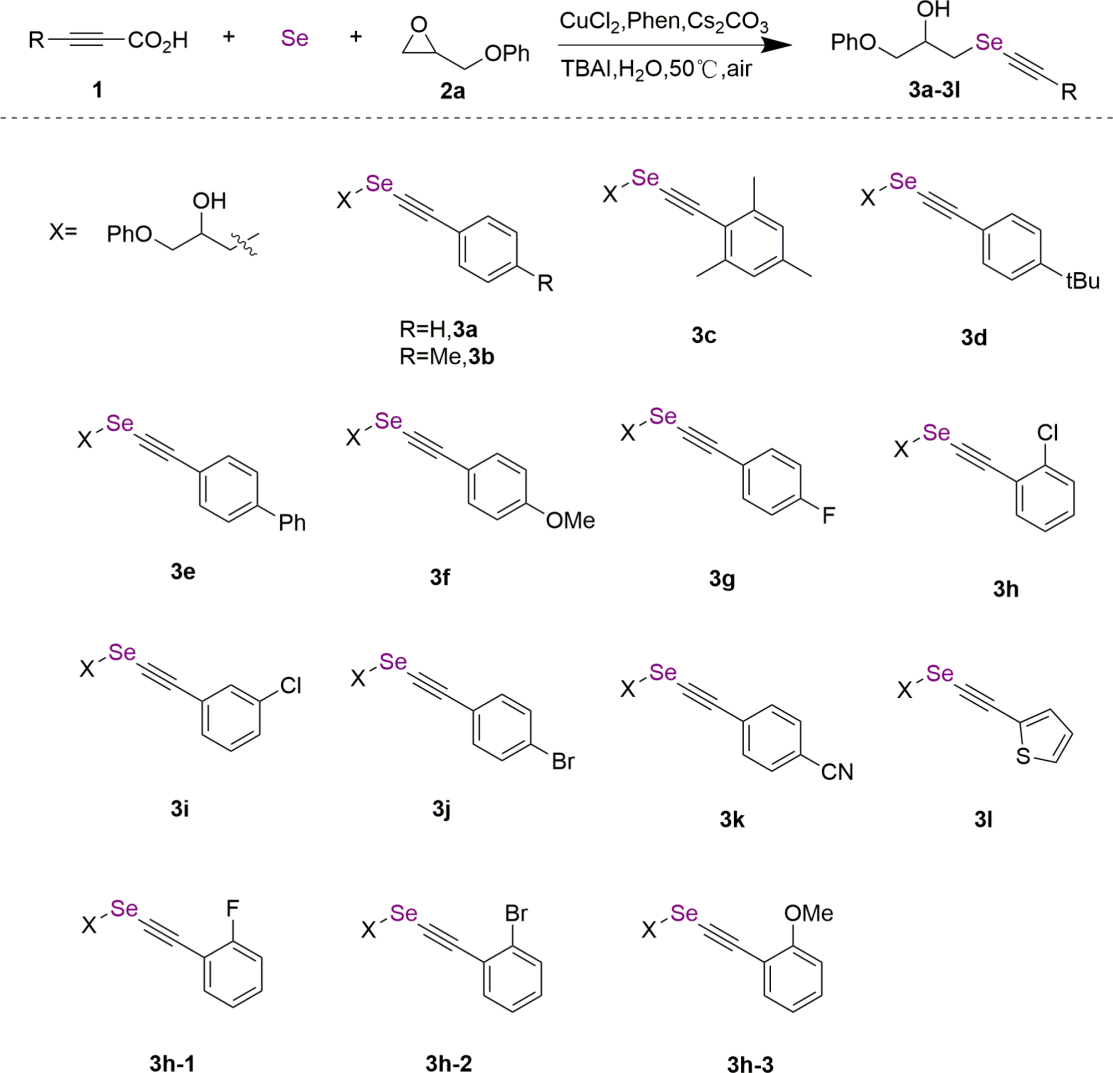
General synthesis of selenium-containing compounds **3a**‒**3L**

The synthesis route of **3a**-**3L** is shown in Fig. 1. Phenylpropionic acid with different substituted functional groups (**1**), Se powder, and glycidylphenyl ether epoxide (**2a**) were used as substrates. CuCl_2_ was used as the catalyst, 1, 10-phen was the efficient ligand, Cs_2_CO_3_ was the base, and TBAI was the phase transfer catalyst in H_2_O at 50 °C to obtain compounds **3a**-**3L** in the yields of 62%~94%. For compounds **3b**-**3k**, they are alkyl (**3b**-**3d**), phenyl (**3e**), methoxy (**3f**), halogen (**3g**-**3j**), and cyano (**3k**) substituted 1-((phenylethynyl)selanyl)-3-phenoxypropan-2-ol, respectively.

**Fig. 2:**
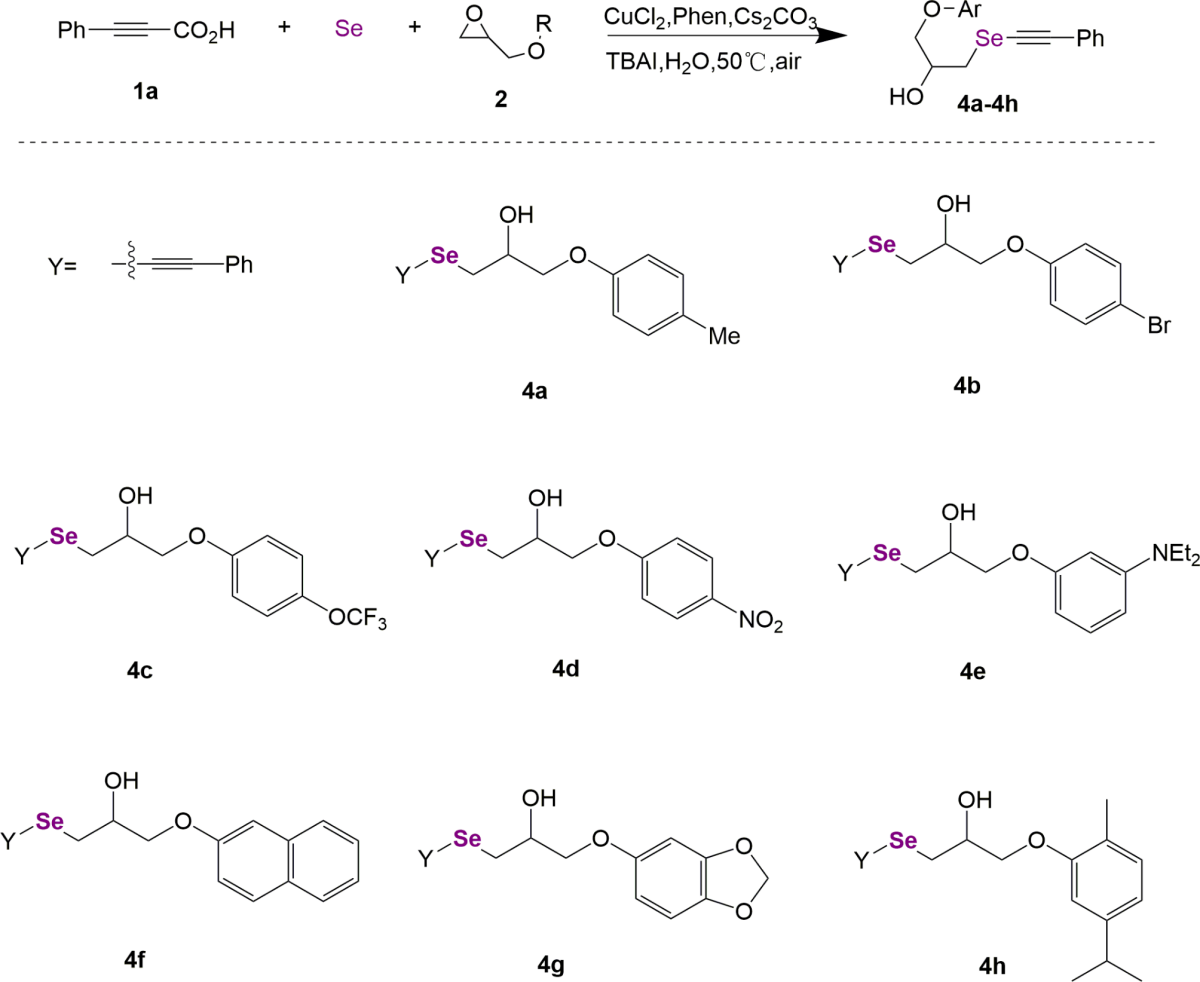
General synthesis of selenium-containing compounds **4a**‒**4h**

The synthesis route of **4a**‒**4h** is shown in Fig. 2. Phenylpropiolic acid (**1a**), Se powder, and various glycidyl phenyl ether epoxides (**2**) were used as reagents. The synthesis conditions were consistent with **3a**-**3L**. Glycidyl phenyl ether epoxides (**2**) were synthesized by reacting various substituted phenols with epichlorohydrin in the presence of phase transfer catalysts and sodium hydroxide. Different substituents of glycidyl phenyl ether epoxides were used to investigate the effects of changes in these functional groups. In the method section, all the synthesized chemical structures were characterized by ESI-MS, ^1^ H NMR, and ^13^ C NMR.

### Structure-activity study

Se-organic compounds reduce the viability of various cancer cell lines [23]. Inspired by these studies, we designed and synthesized two series of structurally related selenium-containing molecules, and their antitumor activity was tested in A549 cells (non-small cell lung carcinoma) [24], HepG2 cells (liver hepatocellular) [25] and RKO cells (human colorectal carcinoma) [26]. A structure-activity relationship analysis based on three cancer cell lines showed that the anti-proliferative activity of **3a**‒**3L** was significantly better than that of **4a**‒**4h**. For compounds **3a**–**3L**, the functional groups alkyl (**3b**‒**3d**), methoxyl (**3f**), halogen (**3g**–**3j**), phenyl (**3e**), and cyano (**3k**) were substituted for aryl propionic acid **1**. We found that arylpropiolic acids bearing electron-deficient substituents (**3g**–**3L**, **3i**–**3k**) showed better activity than those bearing electron-donating substituents (**3a**, **3b**, **3d**, and **3f**). When the phenyl ring carried a methyl group, the anti-proliferation activity of compounds **3a**‒**3c** against cancer cells increased with the number of substituents; compound **3c** showed the most potent anti-proliferation activity. Compound **3d**, a tert-butyl substitution, showed no inhibitory activity, possibly due to its rigid structure. Compounds **3e**‒**3k** contain electron-withdrawing groups. Compounds **3h** and **3g** showed the most potent tumor inhibition activity; their structures are ortho- and meso-substituted phenyls with a chlorine atom, respectively. Thiophene was substituted for phenyl in compound **3L**, which reduced its antitumor activity (Table 1).

**Table 1:** Anti-proliferative activity of compounds **3a**‒**3L** against A549, HepG2, and RKO cell lines

Compound	IC50 (µm)		
	A549	HepG2	RKO
**3a**	>3000	882.00 ± 0.74	119.23 ± 0.44
**3b**	203.10 ± 0.52	65.28 ± 0.24	71.39 ± 0.29
**3c**	52.23 ± 0.43	44.94 ± 0.05	47.60 ± 0.19
**3d**	87.49 ± 0.03	>3000	86.73 ± 0.27
**3e**	40.14 ± 0.87	23.68 ± 0.55	39.48 ± 0.38
**3f**	67.85 ± 0.28	51.38 ± 0.22	40.75 ± 0.41
**3g**	3.17 ± 0.10	5.10 ± 0.18	12.48 ± 1.43
**3h**	0.82 ± 0.14	1.85 ± 0.30	4.20 ± 1.83
**3i**	12.94 ± 0.13	23.40 ± 0.21	19.16 ± 0.22
**3j**	66.00 ± 0.40	47.94 ± 0.44	46.15 ± 0.35
**3k**	55.07 ± 0.25	55.96 ± 0.17	53.13 ± 0.53
**3L**	110.20 ± 0.30	83.29 ± 0.35	98.73 ± 0.63

The anti-proliferative activities of skeleton structure compounds **4a**‒**4h** were also analyzed. None of these compounds had very high antitumor proliferation activity, except for **4c** and **4h** (Table 2).

**Table 2:** Anti-proliferative activity of compounds **4a**‒**4h** against A549, HepG2, and RKO cell lines

Compound	IC50 (µm)		
	A549	HepG2	RKO
**4a**	97.91 ± 0.33	95.35 ± 0.89	97.74 ± 0.27
**4b**	80.00 ± 0.18	75.84 ± 0.56	75.62 ± 0.20
**4c**	59.40 ± 0.10	54.62 ± 0.83	40.31 ± 0.82
**4d**	204.80 ± 0.93	>4000	107.94 ± 0.40
**4e**	88.54 ± 0.14	>50,000	98.14 ± 0.35
**4f**	>20,000	393.00 ± 0.43	80.01 ± 0.29
**4g**	117.80 ± 0.50	>10,000	79.04 ± 0.33
**4h**	53.44 ± 0.44	61.94 ± 0.20	57.99 ± 0.93

Because compound **3h** showed an excellent inhibitory effect in all cancer cell lines, we synthesized compounds **3h-1**, **3h-2**, and **3h-3** to determine whether other electron-absorbing or electron donor groups linked to phenyl would generate a better tumor-inhibiting compound. We found that these derivatives exhibited good antitumor activity, and compound **3h-2** was the best (Table 3). These data suggest that **3h**, **3g**, and **3h-2** have the potential for further investigation. Nonetheless, the antitumor activity of Se compounds synthesized from **3h** was not superior to that of **3h**, suggesting that some core structures with tumor inhibition can be introduced in subsequent structural optimization to enhance antitumor activity [27, 28].

**Table 3:** Anti-proliferative activity of compounds **3h-1**‒**3h-3** against A549, HepG2, and RKO cell lines

Compound	IC50 (µm)		
	A549	HepG2	RKO
**3h-1**	2.77 ± 1.30	2.38 ± 0.43	4.46 ± 0.18
**3h-2**	1.52 ± 0.16	2.22 ± 0.32	1.56 ± 0.98
**3h-3**	3.20 ± 0.25	3.49 ± 0.11	2.87 ± 0.44

### Effect of compounds 3h, 3g, and 3h-2 on A549 cell migration

**Fig. 3:**
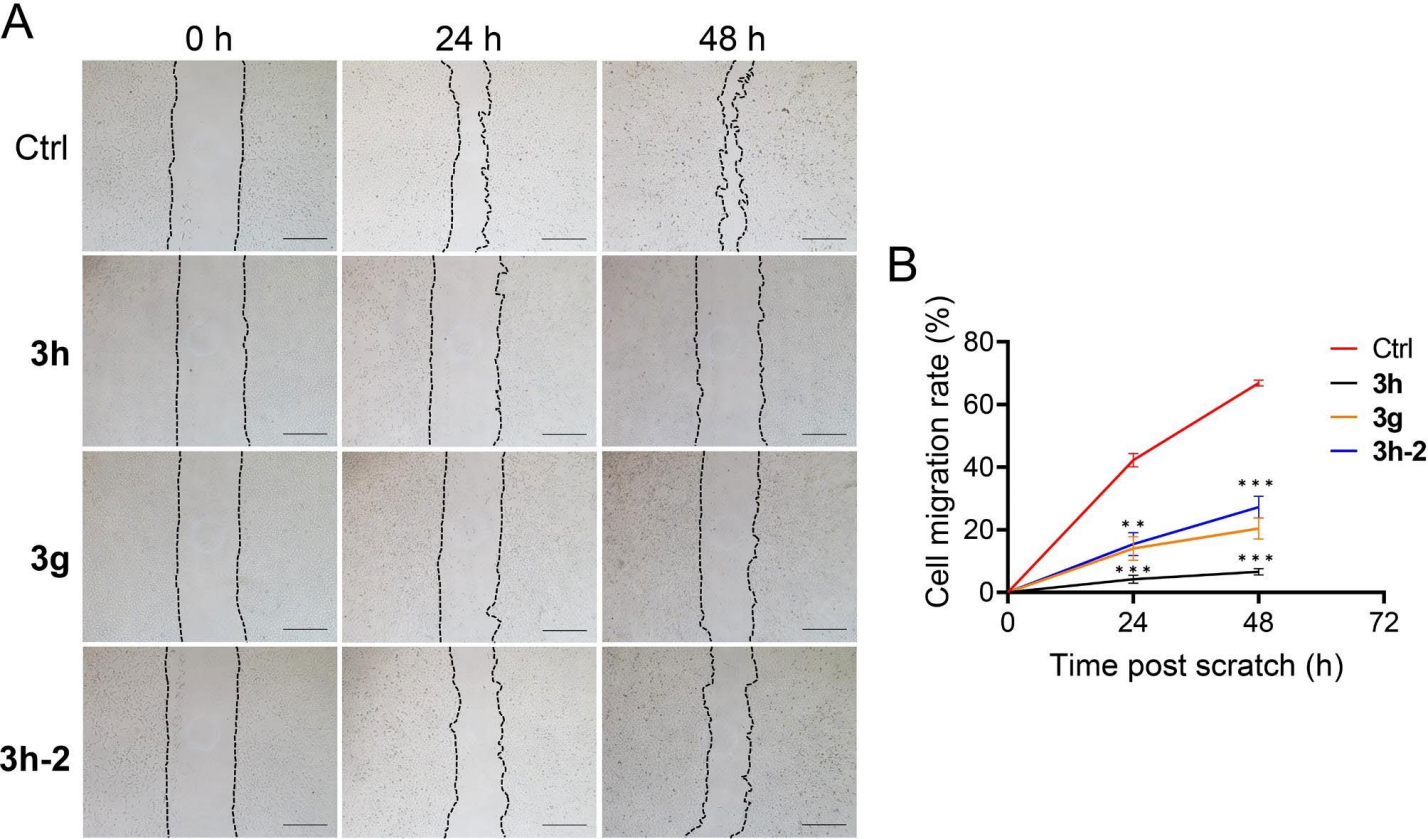
Effect of **3h**, **3g**, and **3h-2** on the migration of A549 cells. **(A**, **B**) A549 cells treated with compound **3h**, **3g**, and **3h-2** (1 µmol) in free serum DMEM for 48 h. (**A**) Representative images of A549 cells at 0 h, 24 h, and 48 h post scratch (*n* = 5). Scale bars = 50 μm. (**B**) The quantitative ratio of the cell migration area to the scratch area at 0 h. All data are expressed as mean ± SEM. ^***^p < 0.001, ^**^p < 0.01 compared to 0 h

Since compounds **3h**, **3g**, and **3h-2** showed suitable anti-proliferative activities against cancer cells, we measured the ability of **3h**, **3g**, and **3h-2** to inhibit cell migration. Typical images were taken at the beginning of the experiment (0h) and after treatment with compounds or vehicle for 24 or 48 h. Compounds **3h**, **3g**, and **3h-2** inhibited the migration ability of A549 cells in a time-dependent manner (Fig. 3A). The cell migration rate was decreased by 60.2% after treatment with 1 µmol of **3h** for 48 h. The same result was observed after treatment with **3g** and **3h-2**, with the cell migration rate decreasing by 46.4% and 39.6%, respectively (Fig. 3B>). These results suggest that **3h**, **3g**, and **3h-2** inhibit the migration and invasion of A549 cells and may be chemotherapy agents for metastatic cancer.

### Effect of compounds 3h, 3g, and 3h-2 on A549 cell apoptosis using Annexin V-FITC/PI

**Fig. 4:**
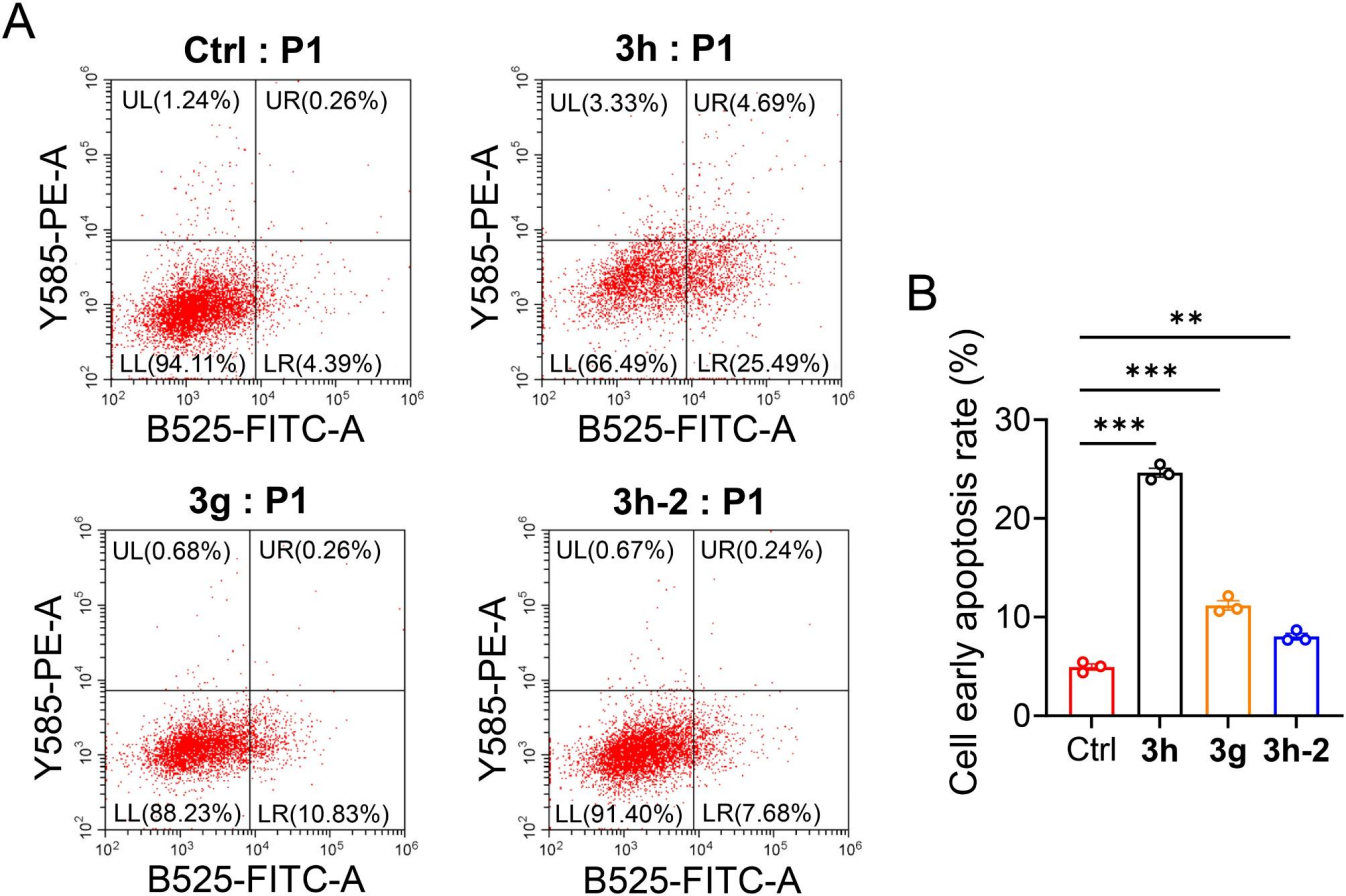
Apoptosis analysis of A549 lung tumor cells using flow cytometry. (**A**) Dot plot representation of annexin-V-FITC fluorescence (X-axis) vs. PI fluorescence (Y-axis) of apoptotic A549 (annexin-V positive) cells treated with **3h**, **3g**, and **3h-2** (1 µmol) for 12h (*n* = 3). (**B**) Early apoptotic cell percentages were obtained by EXPO32 ADC analysis software. All data are expressed as mean ± SEM. ^***^p < 0.001, ^**^p < 0.01 compared to the control group

We conducted several experiments in vitro to determine whether the tumor inhibition effect was mediated by apoptosis. The ability of compounds **3h**, **3g**, and **3h-2** to induce apoptosis was evaluated by measuring apoptosis using flow cytometry in human cancer cells. Treating A549 cells with **3h**, **3g**, and **3h-2** at 1 µmol for 12h led to early-stage cell apoptosis at 25.49%, 10.83%, and 7.68%, respectively. There was only 4.39% apoptosis in untreated cells (Fig. 4A and B). This finding suggests that the inhibition of cell proliferation mediated by **3h**, **3g**, and **3h-2** is related to increased apoptosis, especially for **3h**.

### Effect of compounds 3h, 3g, and 3h-2 on cancer and normal cells

**Fig. 5:**
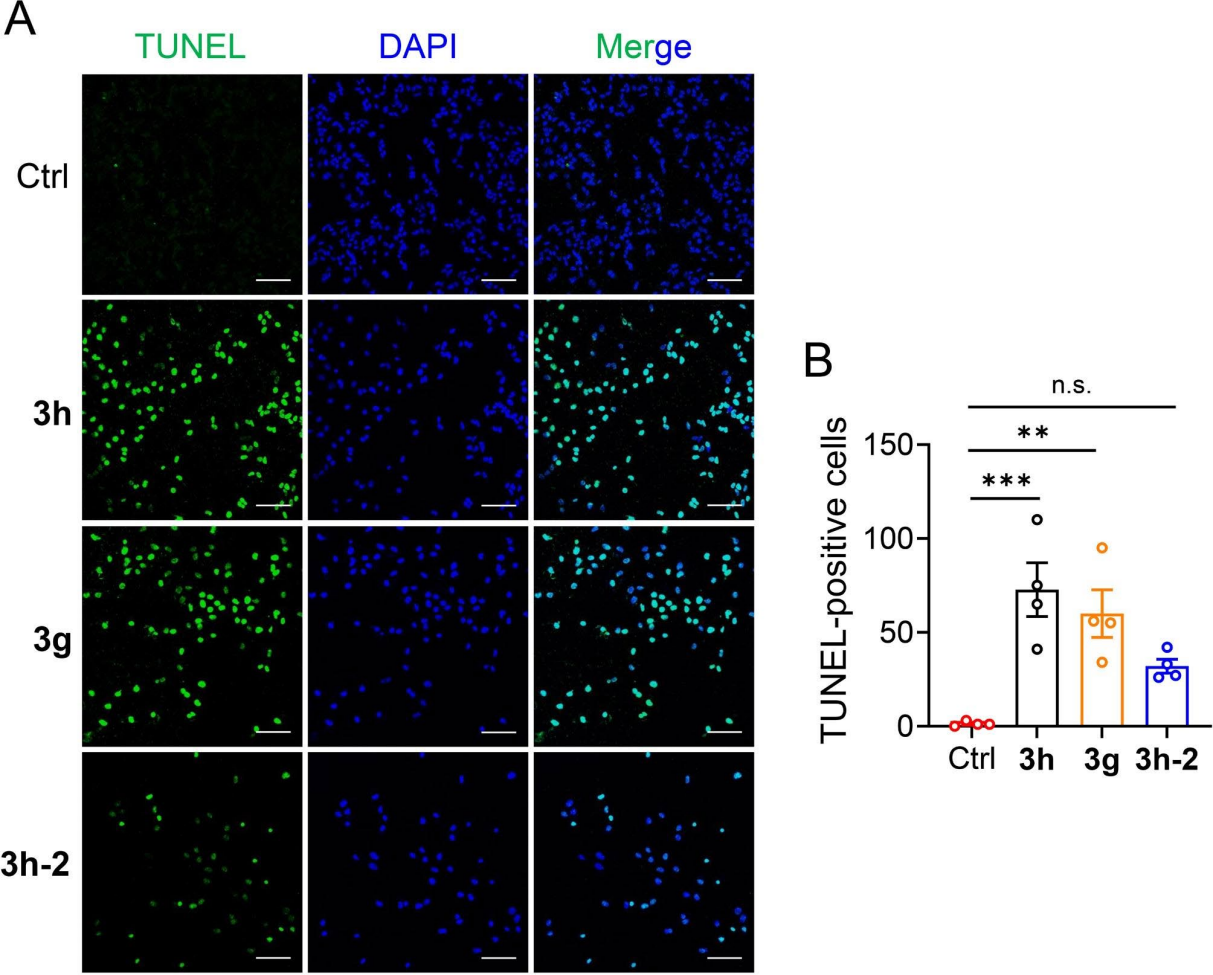
TUNEL staining of compounds **3h**, **3g**, and **3h-2** in A549 cells. (**A**) Representative images of TUNEL staining of A549 cells treated with compounds **3h**, **3g**, and **3h-2** at 1 µmol concentration for 24 h (*n* = 4). Scale bars = 100 μm. (**B**) Quantification of TUNEL-positive cells in panel **A**. All data are expressed as mean ± SEM. ^***^p < 0.001, ^**^p < 0.01 compared to the control group; n.s., not significant

TUNEL staining showed that A549 cells treated with **3h**, **3g**, and **3h-2** exhibited more significant numbers of apoptotic cells with fragmented nuclei than the control (Fig. 5A). The order of TUNEL-positive cells was as follows: **3h** > **3g** > **3h-2** > Control (Fig. 5B). These results suggest that compounds **3h**, **3g**, **3h-2**, and (especially) **3h** inhibit cell proliferation and promote apoptosis in A549 cells.

To investigate whether these compounds are active against normal cells, we conducted a toxicity experiment on podocyte cells (MPC) and hepatocyte cells (AML-12), which are two normal cell types involved in drug metabolism. The MTT results indicated that the three representative compounds exhibited almost no toxic side effects on normal liver cells and renal tubular cells even at a concentration of 10 µM, far exceeding the IC50 value of the compounds for cancer cell lines (Supplementary Fig. S3).

### Mechanistic study of compound 3h on A549 cells at the protein level

**Fig. 6:**
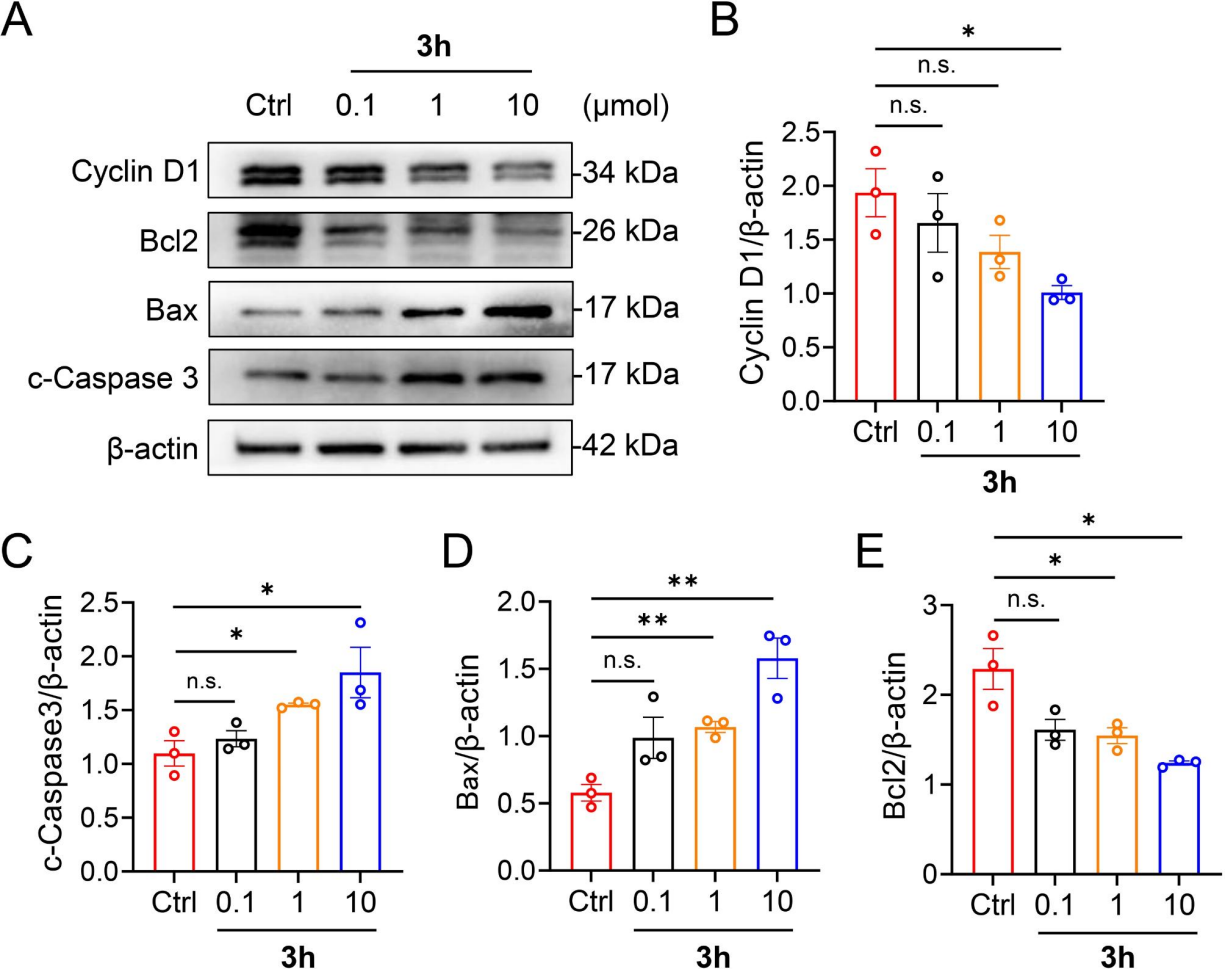
Cell signaling in A549 cells treated with **3h** at different concentrations (0.1, 1, and 10 µmol) for 24h. (**A**) Western blotting analysis of the expression of Cyclin D1, Bcl2, Bax, and cleaved Caspase 3 (*n* = 3). β-actin was used as a loading control. (**B**-**E**) Statistical analysis of the relative protein intensity of (**B**) Cyclin D1, (**C**) cleaved Caspase 3, (**D**) Bax, and (**E**) Bcl2 in A549 cells. Full-length gels are presented in Supplementary Fig. S1. All data are expressed as mean ± SEM. ^*^p < 0.05, ^**^p < 0.01 compared to the control group; n.s., not significant

Cyclin D1 promotes cell proliferation by promoting the cell cycle transition from G1 to S phase via binding and activating CDK4, a Cyclin-dependent kinase specific to the G1 phase. The abundance of Cyclin D1 was less than that of the control after treatment with compound **3h** at 1 µmol (Fig. 6A and B).

The effect of compound **3h** on apoptosis was also tested. After treatment with compound **3h**, the pro-apoptotic proteins cleaved Caspase 3 and Bax were dose-dependently upregulated, while the anti-apoptotic protein Bcl2 was downregulated in A549 cells (Fig. 6A C-E).

### Proliferation and apoptosis-related mRNA levels

**Fig. 7:**
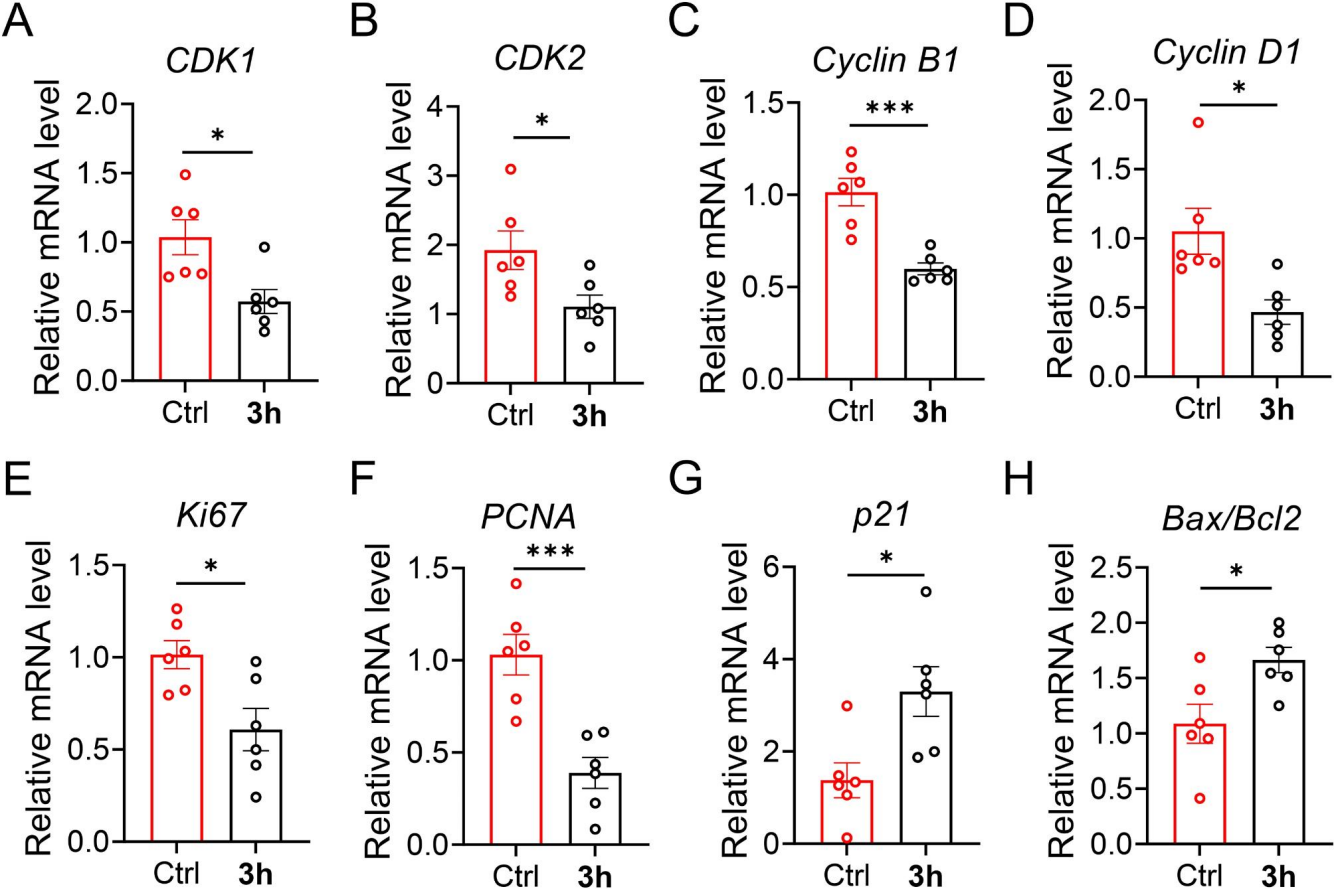
Relative mRNA levels of (**A**) *CDK1*, (**B**) *CDK2*, (**C**) *Cyclin B1*, (**D**) *Cyclin D1*, (**E**) *Ki67*, (**F**) *PCNA*, (**G**) *p21*, and (**H**) *Bax*/*Bcl2* genes in the A549 cells that were treated with **3h** at 1 µmol concentrations or PBS control for 24 h. Relative mRNA levels were normalized to that of *β-actin*. The data were from six independent measurements. All data are expressed as mean ± SEM. ^***^p < 0.001, ^*^p < 0.05 compared to the control group

We then investigated the correlation of **3h**, which induced cell cycle arrest, with alterations in the expression of proteins that regulate cell division. CDK1, CDK2, and Cyclin B1 induce eukaryotic cells to enter mitosis. We measured the relative mRNA levels and found that CDK1, CDK2, and Cyclin B1 mRNA were decreased after treatment with compound **3h** (Fig. 7A-C). Real-time PCR quantification revealed that Cyclin D1 levels were markedly higher in the control group than in the **3h-**treated group (Fig. 7D). The mRNA levels of the cell proliferation-related proteins Ki67 and PCNA showed the same trend (Fig. 7E and F).

p21 is a member of the cyclin-dependent kinase inhibitor family. It is associated with tumor inhibition and inhibits CDK activity. qRT‒PCR quantification revealed that the expression of p21 was markedly higher in the **3h-**treated group than in the control group (Fig. 7G). These results suggest that compound **3h** induces cell cycle arrest and reduces cell proliferation in A549 cells. In addition, Bax/Bcl2 mRNA levels were significantly increased after treatment with compound **3h** at 1 µmol (Fig. 7H).

### Antitumor activity of compound 3h in a xenograft tumor model

**Fig. 8:**
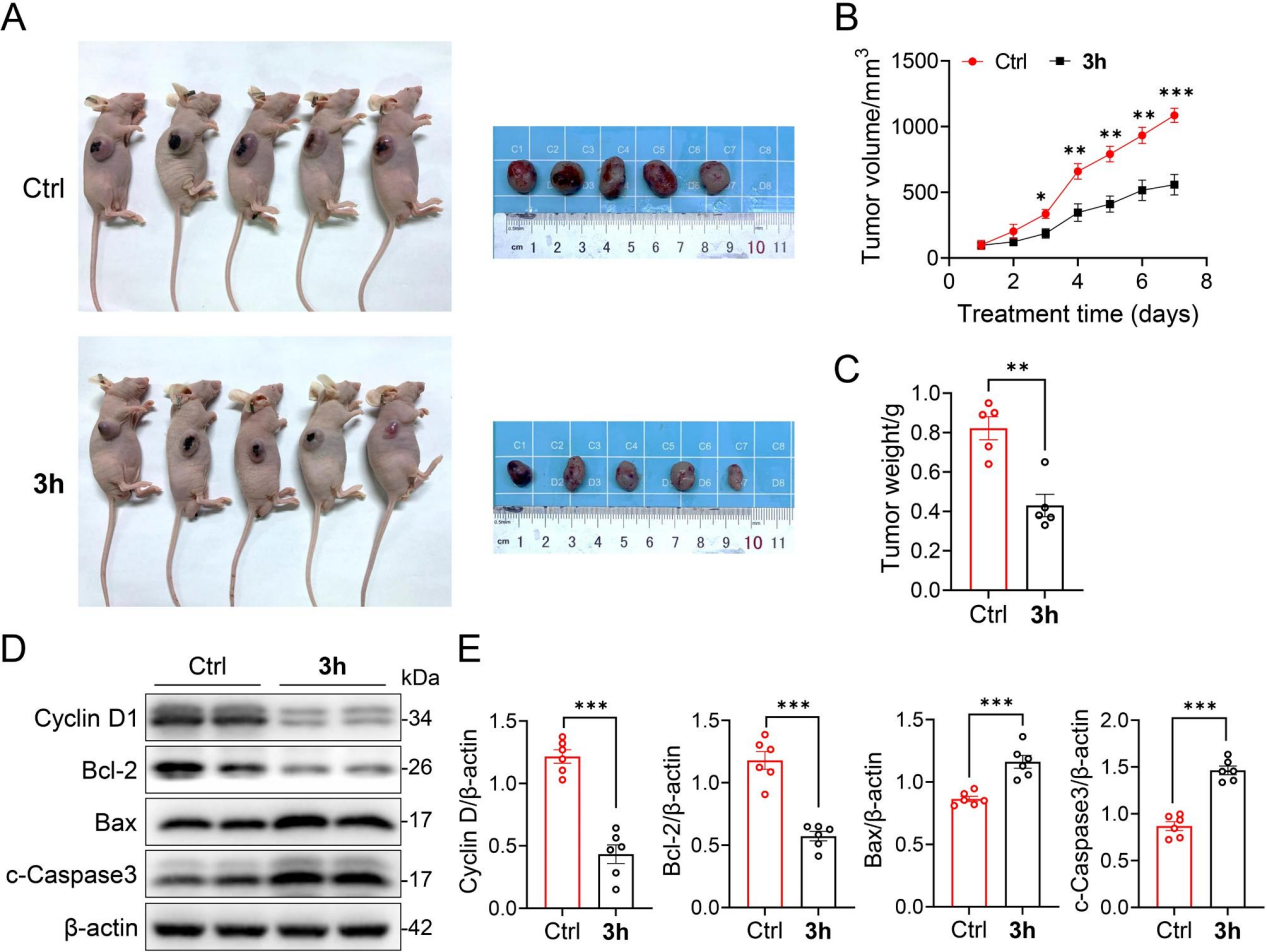
In vivo antitumor effect of compound **3h**. (**A-E**) A549 cells were injected into the flanks of nude mice. When the tumor volume reached approximately 100 mm^3^, the nude mice were sorted into two groups (*n* = 5) and one group was intraperitoneally injected with **3h** (2.5mg/kg, once a day) for 14 days. (**A**) Images of sacrificed mice and excised tumors in each group. (**B**) The development of tumor size during the treatment period was recorded. (**C**) At the end of the experiment, tumors were resected and weighed. (**D**) Western blotting analysis of the expression of Cyclin D1, c-Caspase3, Bax, and Bcl2 in total tumor lysates from vehicle- and **3h**-treated mice (*n* = 3). β-actin was used as a loading control. (**E**) Expression levels were quantitated using ImageJ software. Full-length gels are presented in Supplementary Fig. S2. All data are expressed as mean ± SEM. ^***^p < 0.001, ^**^p < 0.01, ^*^p < 0.05 compared to the control group

A xenograft model was established by subcutaneous injection of A549 cells at the logarithmic growth phase into the right axilla of mice. When the tumor size reached approximately 100 mm^3^, the mice were randomly divided into two groups (PBS-treated and **3h**-treated groups), with five mice per group. The mice were intraperitoneally injected with compound **3h** at 2.5 mg/kg daily during the observation period. After treatment for 14 days, the tumor was excised and weighed. The mean tumor volume in the PBS-treated group was 1.94 times that of the **3h**-treated group (Fig. 8A and B). Treatment with **3h** significantly reduced tumor size and weight (Fig. 8C). In addition, we also evaluated the impact of **3h** on the growth and apoptosis of mouse tumors using Western blotting. Compared with the control group, the expression of Cyclin D1 in total tumor lysates from mice after **3h** treatment was significantly decreased, while the pro-apoptotic proteins c-Caspase3 and Bax were upregulated and the anti-apoptotic protein Bcl2 was downregulated (Fig. 8D and E). Although compound **3h** exerts strong antitumor activity, the specific mechanisms and targets of this series of compounds still need to be further elucidated.

### Protection of compound 3h against cisplatin-induced damage to normal cells

**Fig. 9:**
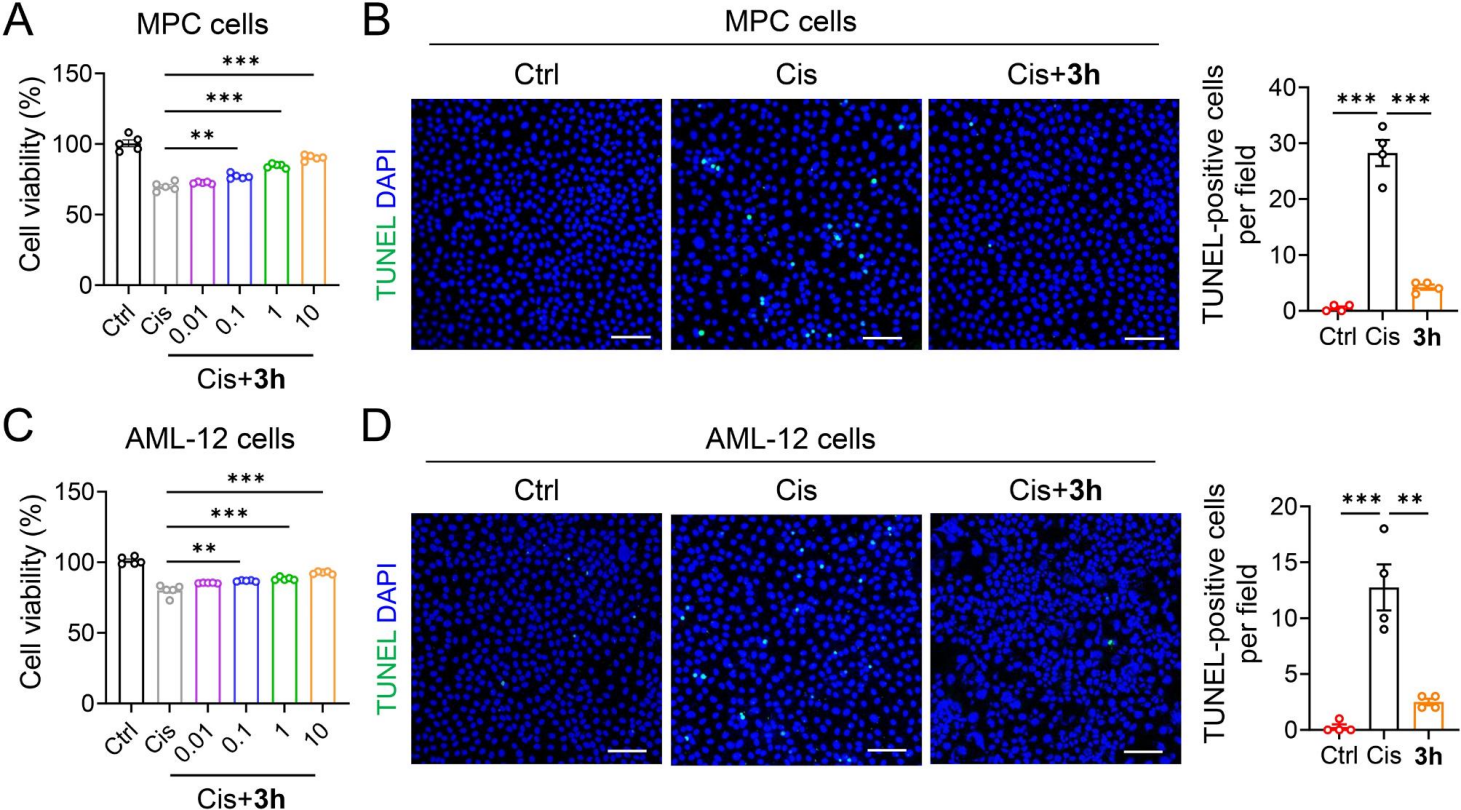
MTT assay and TUNEL staining in MPC and AML-12 cells. (**A**) MPC cells were cultured with 20 µmol cisplatin or 20 µmol cisplatin combined with 0.01, 0.1, 1, and 10 µmol **3h** for 12 h, and cell viability was determined by MTT test. (**B**) Representative images and quantification of TUNEL staining of MPC cells treated with 20 µmol cisplatin or 20 µmol cisplatin combined with 1 µmol **3h** for 12 h. Scale bars = 100 μm. (**C**) AML-12 cells were cultured with 20 µmol cisplatin or 20 µmol cisplatin combined with 0.01, 0.1, 1, 10 µmol **3h** for 12 h, and cell viability was determined by MTT assay. (**D**) Representative images and quantification of TUNEL staining of AML-12 cells treated with 20 µmol cisplatin or 20 µmol cisplatin and 1 µmol **3h** for 24h. Scale bars = 100 μm. *n* = 4 per group. All data are expressed as mean ± SEM. ^***^p < 0.001, ^**^p < 0.01 compared to the Cis group

Nephrotoxicity and hepatotoxicity are significant dose-limiting side effects in cisplatin-based chemotherapy [29, 30]. Se compounds protect normal cells from damage. Considering the organoselenium compounds have synergistic effects when combined with chemotherapeutic drugs, we explored the protective activity of **3h** against cisplatin-induced MPC and AML-12 cells. As shown in Fig. 9A and C, the viability of MPC and AML-12 cells was reduced after treatment with cisplatin, which was significantly increased in a dose-dependent manner after **3h** treatment. TUNEL results showed that the number of positive cells significantly decreased after **3h** treatment compared with the cisplatin-induced group (Fig. 9B and D). These findings suggest that compound **3h** has a protective effect on normal cells, and a synergistic effect can be achieved when combined with cisplatin.

## Conclusion

Selenocompounds promote cell death via apoptosis and protect cells against oxidative stress-induced death. The anticancer effect of Se might be mediated by the prevention of damage to healthy cells and apoptosis of tumor cells [31]. In the present study, two series of novel phenoxy-((phenylethynyl) selanyl) propan-2-ol derivatives were synthesized, and their anti-proliferation activities were evaluated. These compounds exhibited intense anti-proliferation activity against three human cancer cells, with IC50 values in the submicromolar concentration range. Compounds **3h**, **3g**, and **3h-2** exhibited the best activity against cancer cells. Further flow cytometry analysis showed that **3h**, **3g**, and **3h-2** induced G2/M phase arrest and apoptosis of A549 cells. Cellular studies showed that apoptosis induction by **3h** was associated with expression changes in several cell cycle-related proteins (e.g., Cyclin B1, Cyclin D1, CDK1, and CDK2) and apoptosis-related proteins (e.g., Bcl-2, Bax, and cleaved-Caspase 3). After treatment with compound **3h**, the level of cleaved caspase 3 and the ratio of Bax to Bcl-2 protein level significantly increased. The xenograft tumor experiment in nude mice revealed that compound **3h** has antitumor activity in vivo without evident toxicity. Compound **3h** protected against cisplatin-induced injury to normal cells. These in vitro and in vivo findings suggest that the 1-phenoxy-3-((phenylethynyl) selanyl) propan-2-ol derivative **3h** is a promising lead compound for developing anticancer drugs.

### Electronic supplementary material

Below is the link to the electronic supplementary material.

Supplementary Material 1

## Data Availability

All data generated or analyzed during this study are included in this published article.
