# Supplementary material for: Novel phenoxy-((phenylethynyl) selanyl) propan-2-ol derivatives as potential anticancer agents
Source: BMC Chem. 2023 Nov 28;17(1):172. doi: 10.1186/s13065-023-01076-0 (PMC10685490; doi:10.1186/s13065-023-01076-0)
Supplement: Supplementary file 1 — Supplementary Material 1 [file 13065_2023_1076_MOESM1_ESM.docx]

**Supplementary Information**

**Novel 1-phenoxy-3-((phenylethynyl) selanyl) propan-2-ol derivatives as potential anticancer agents**

Wenxin Xu^1, #^, Yali Du^1, 2 #^, Beibin Pan^1, #^, Qiying Wang^1^, Haoran Zheng^1^, Ruonan Zhang^2^, Jiaxin Lou^1^, Guanghui Zhu^3, *^, Jie Zhou^1, *^, Jian Sun^1, *^

^1^ School of Pharmaceutical Sciences, Wenzhou Medical University, Zhejiang, China

^2^ Sir Run Run Shaw Hospital, School of Medicine, Zhejiang University

^3^ The Second Affiliated Hospital and Yuying Children’s Hospital of Wenzhou Medical University, Zhejiang, China

^#^ These authors contributed equally to this work.

^*^ To whom correspondence should be addressed.

E-mail: sunjian@wmu.edu.cn; zhoujie.0628@163.com; lcylhy@126.com

**Table S1** Primer sequences for qRT-PCR

| **Gene Name** | **Sequence (5’-3’)** |
| --- | --- |
| Human-*CDK1* | Forward: 5’-GGATGTGCTTATGCAGGATTCC-3’ |
|  | Reverse: 5’-CATGTACTGACCAGGAGGGATAG-3’ |
| Human-*CDK2* | Forward: 5’-CCAGGAGTTACTTCTATGCCTGA-3’ |
|  | Reverse: 5’-TTCATCCAGGGGAGGTACAAC-3’ |
| Human-*Cyclin D1* | Forward: 5’-GCTGCGAAGTGGAAACCATC-3’ |
|  | Reverse: 5’-CCTCCTTCTGCACACATTTGAA-3’ |
| Human-*Cyclin B1* | Forward: 5’-AACTTTCGCCTGAGCCTATTTT-3’ |
|  | Reverse: 5’-TTGGTCTGACTGCTTGCTCTT-3’ |
| Human-*p21* | Forward: 5’-TGTCCGTCAGAACCCATGC-3’ |
|  | Reverse: 5’-AAAGTCGAAGTTCCATCGCTC-3’ |
| Human-*Ki67* | Forward: 5’-ACGCCTGGTTACTATCAAAAGG-3’ |
|  | Reverse: 5’-CAGACCCATTTACTTGTGTTGGA-3’ |
| Human-*PCNA* | Forward: 5’-GCGTGAACCTCACCAGTATGT-3’ |
|  | Reverse: 5’-TCTTCGGCCCTTAGTGTAATGAT-3’ |
| Human-*Bax* | Forward: 5’-CCCGAGAGGTCTTTTTCCGAG-3’ |
|  | Reverse: 5’-CCAGCCCATGATGGTTCTGAT-3’ |
| Human-*Bcl2* | Forward: 5’-GGTGGGGTCATGTGTGTGG-3’ |
|  | Reverse: 5’-CGGTTCAGGTACTCAGTCATCC-3’ |
| Human-*β-actin* | Forward: 5’-GAAGATCAAGATCATTGCTCCTC-3’ |
|  | Reverse: 5’-ATCCACATCTGCTGGAAGG-3’ |


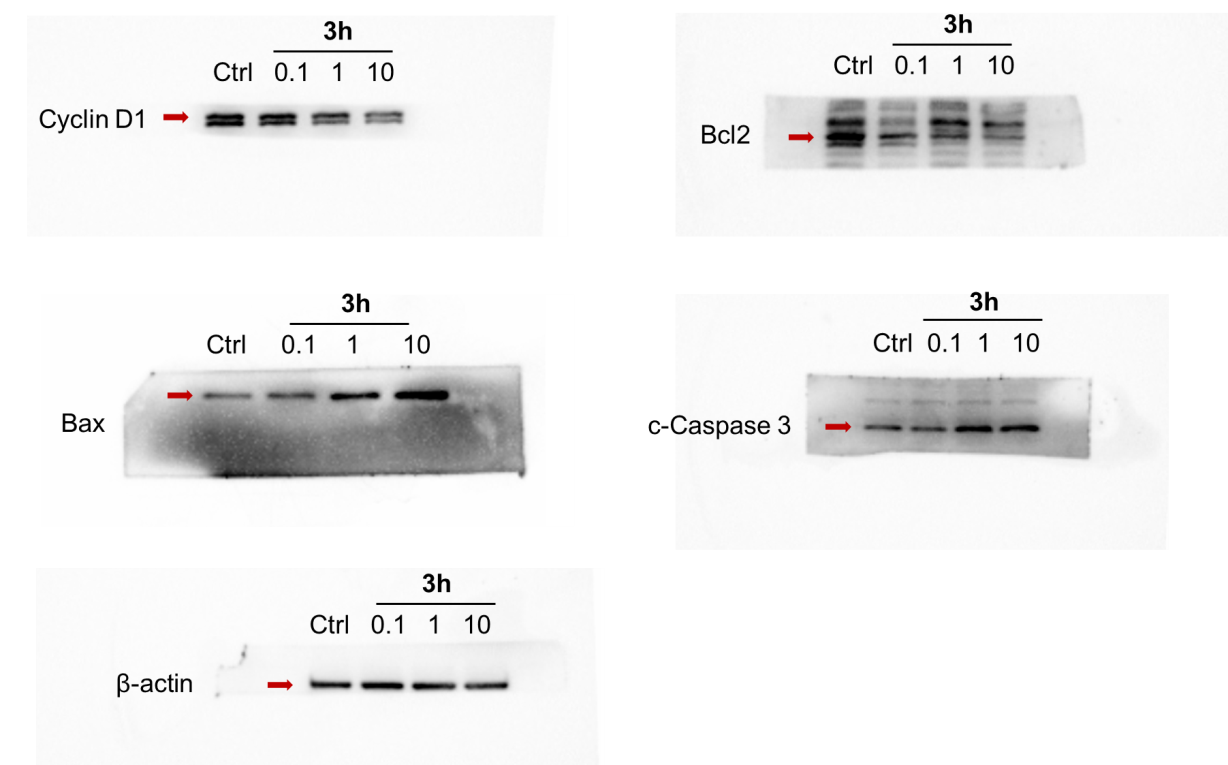


**Fig. S1** Full-length gels for the Fig. 6.


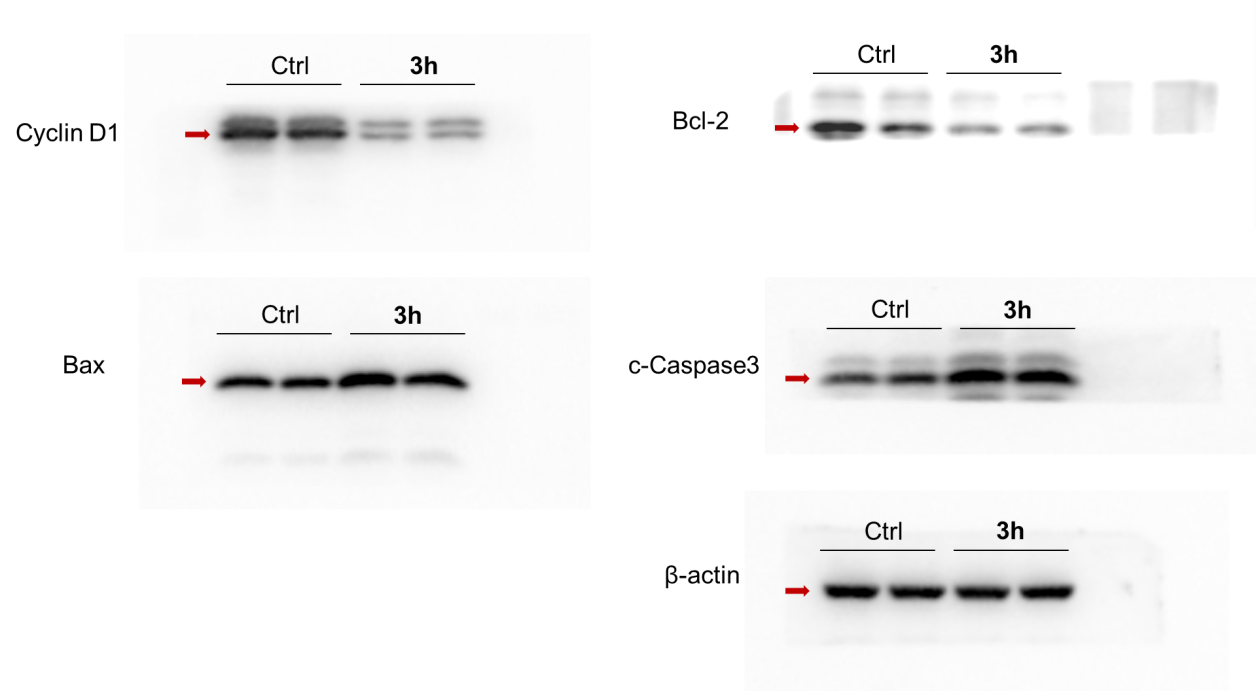
 **Fig. S2** Full-length gels for the Fig. 8.


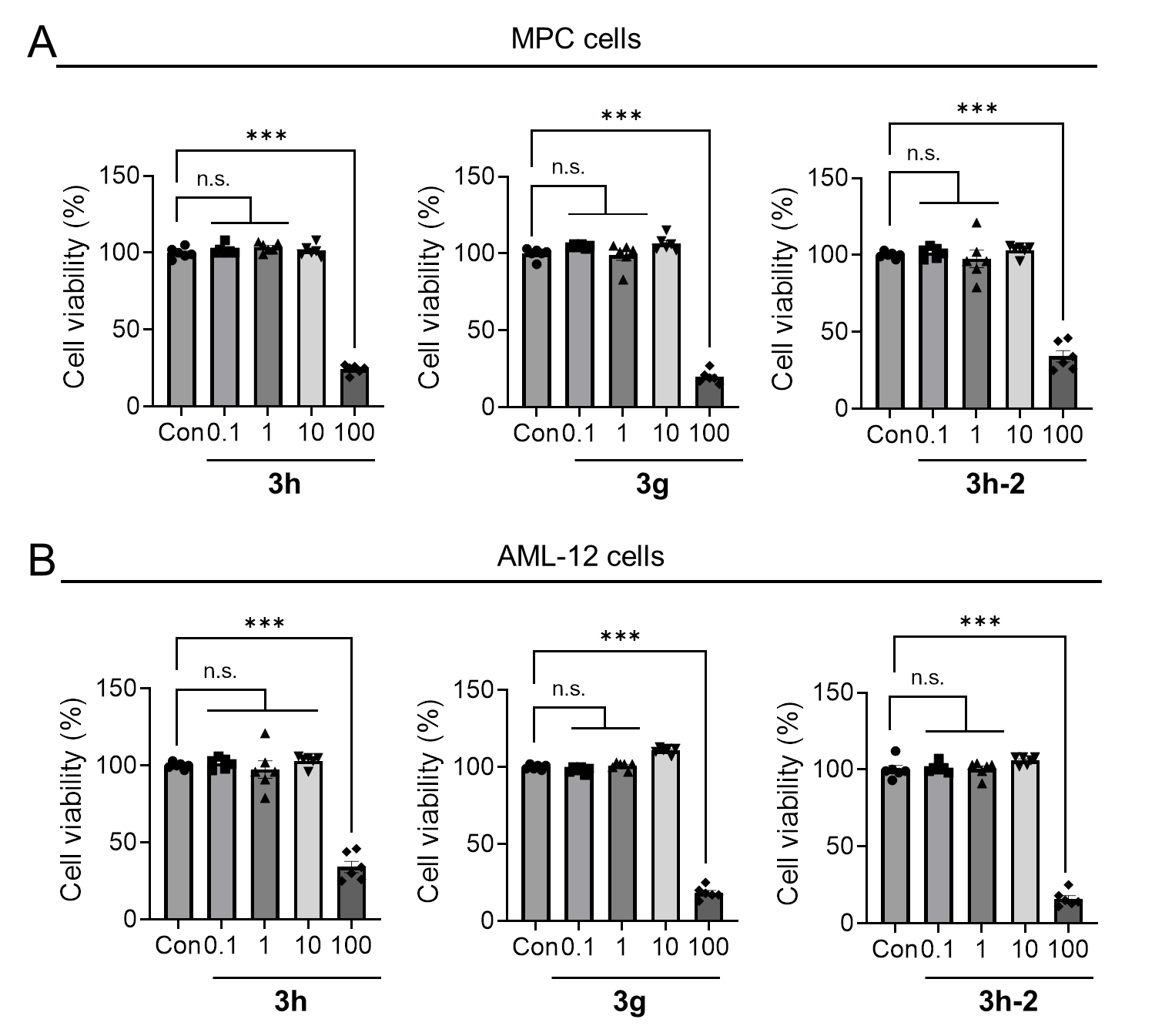


**Fig. S3** Effect of **3h**, **3g**, and **3h-2** on cell viability of MPC and AML-12 cells. (**A**) MPC cells and (**B**) AML-12 cells were cultured with 0.01, 0.1, 1, and 10 μmol **3h**, **3g**, or **3h-2** for 24 h, and cell viability was determined by MTT assay. All data are expressed as mean ± SEM. ^***^p < 0.001 compared to the control group; n.s., not significant.


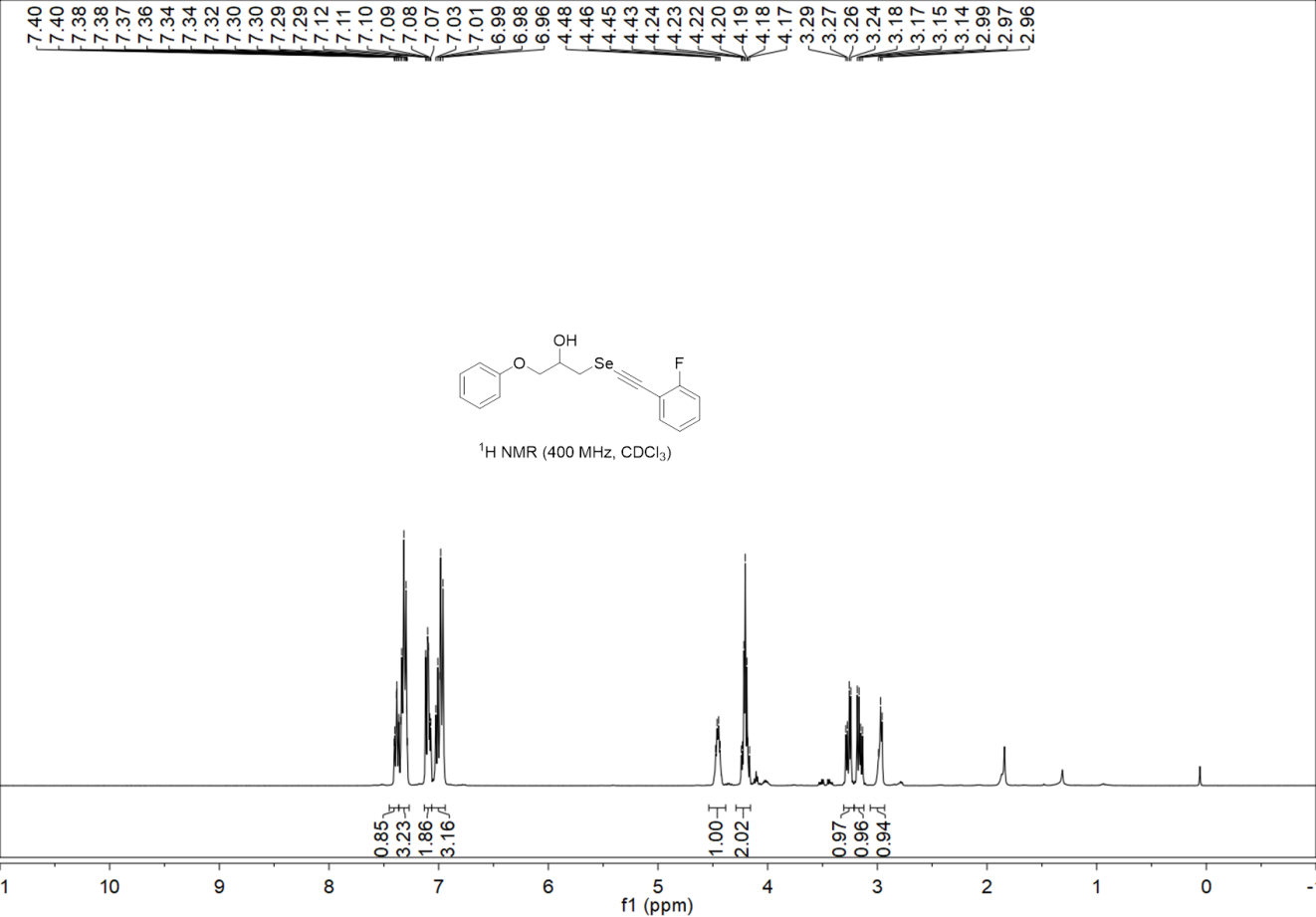


**Fig. S4** ^1^H NMR spectrum of compound **3h-1**


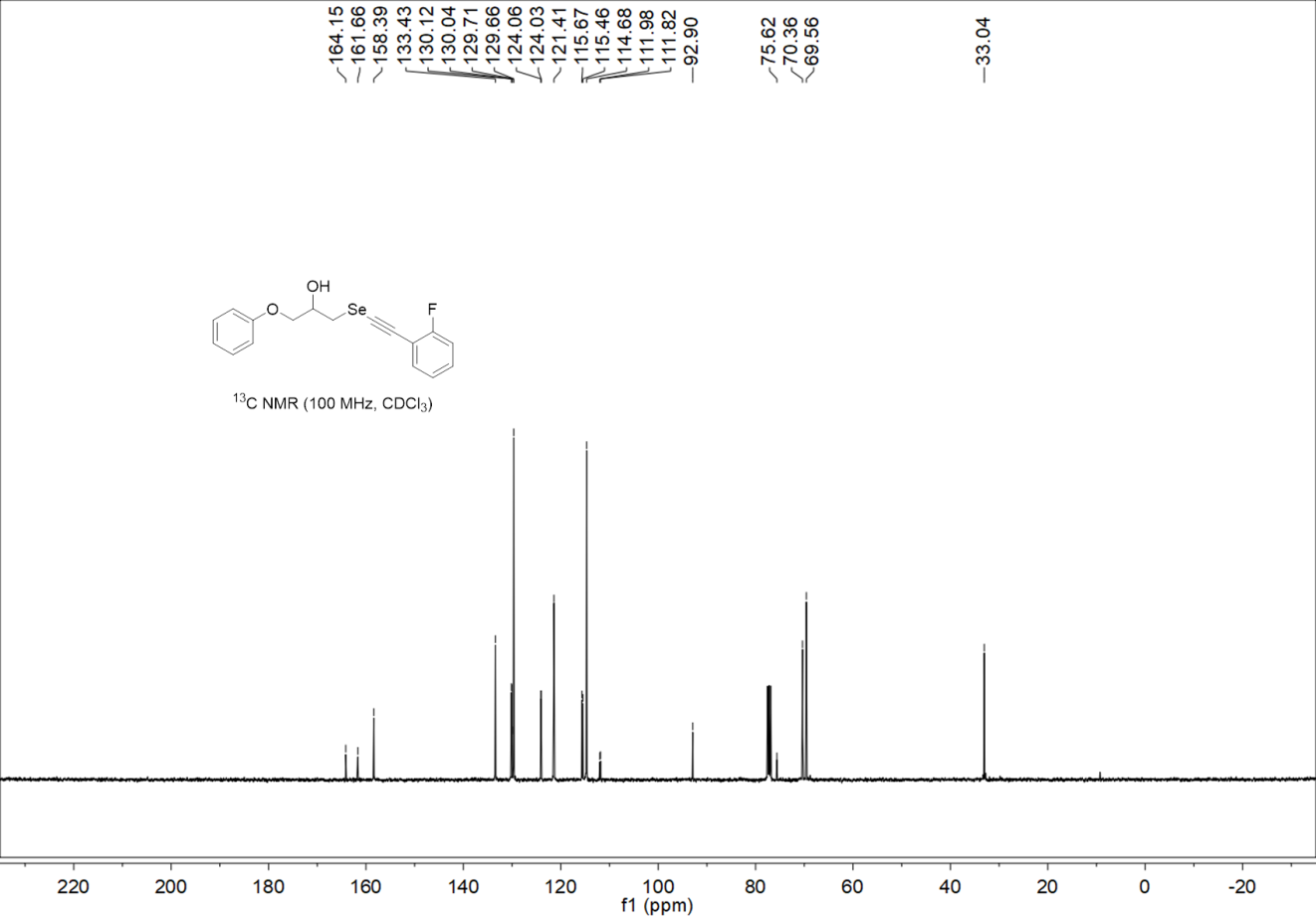


**Fig. S5** ^13^C NMR spectrum of compound **3h-1**

**
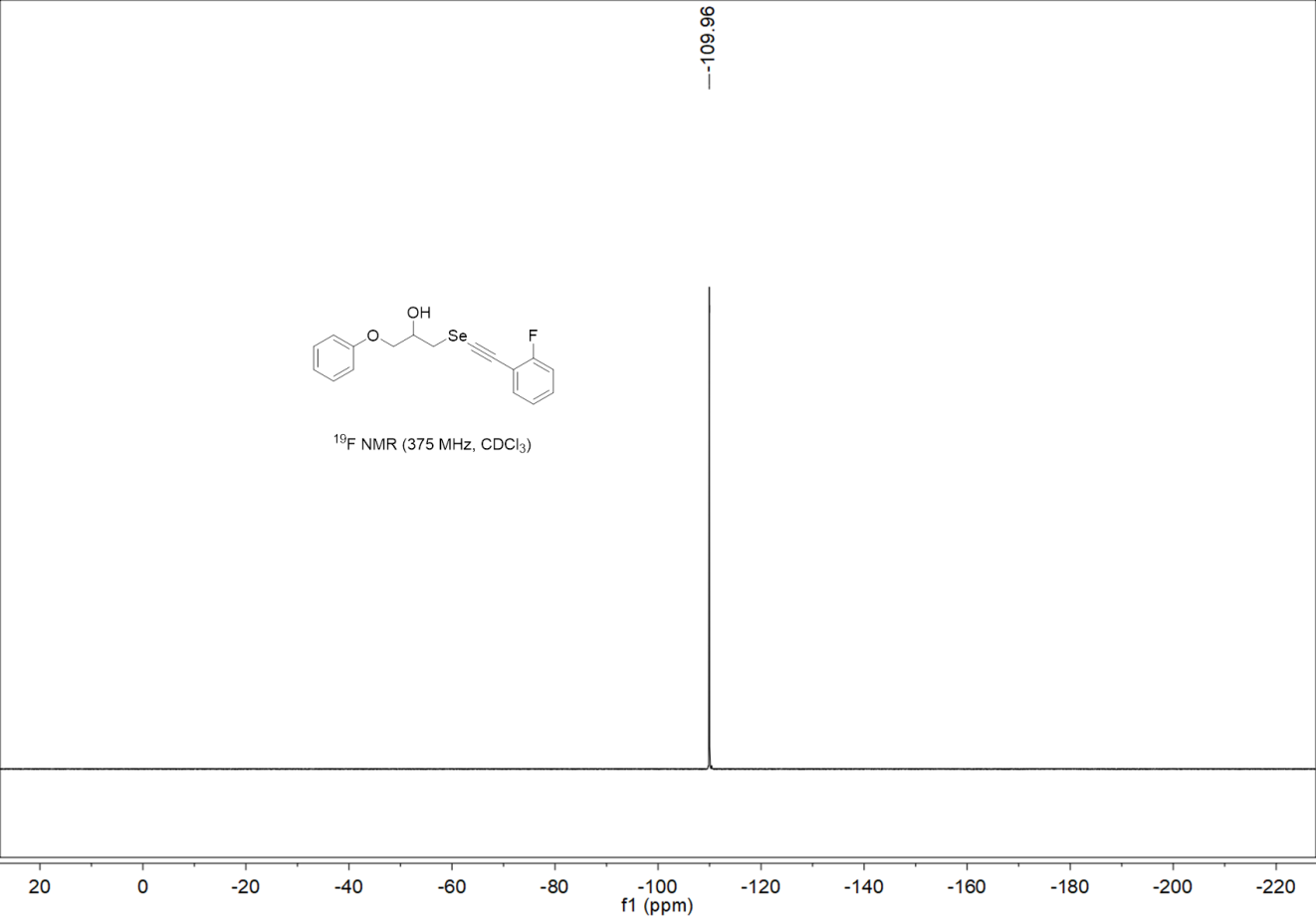
**

**Fig. S6** ^19^F NMR spectrum of compound **3h-1**


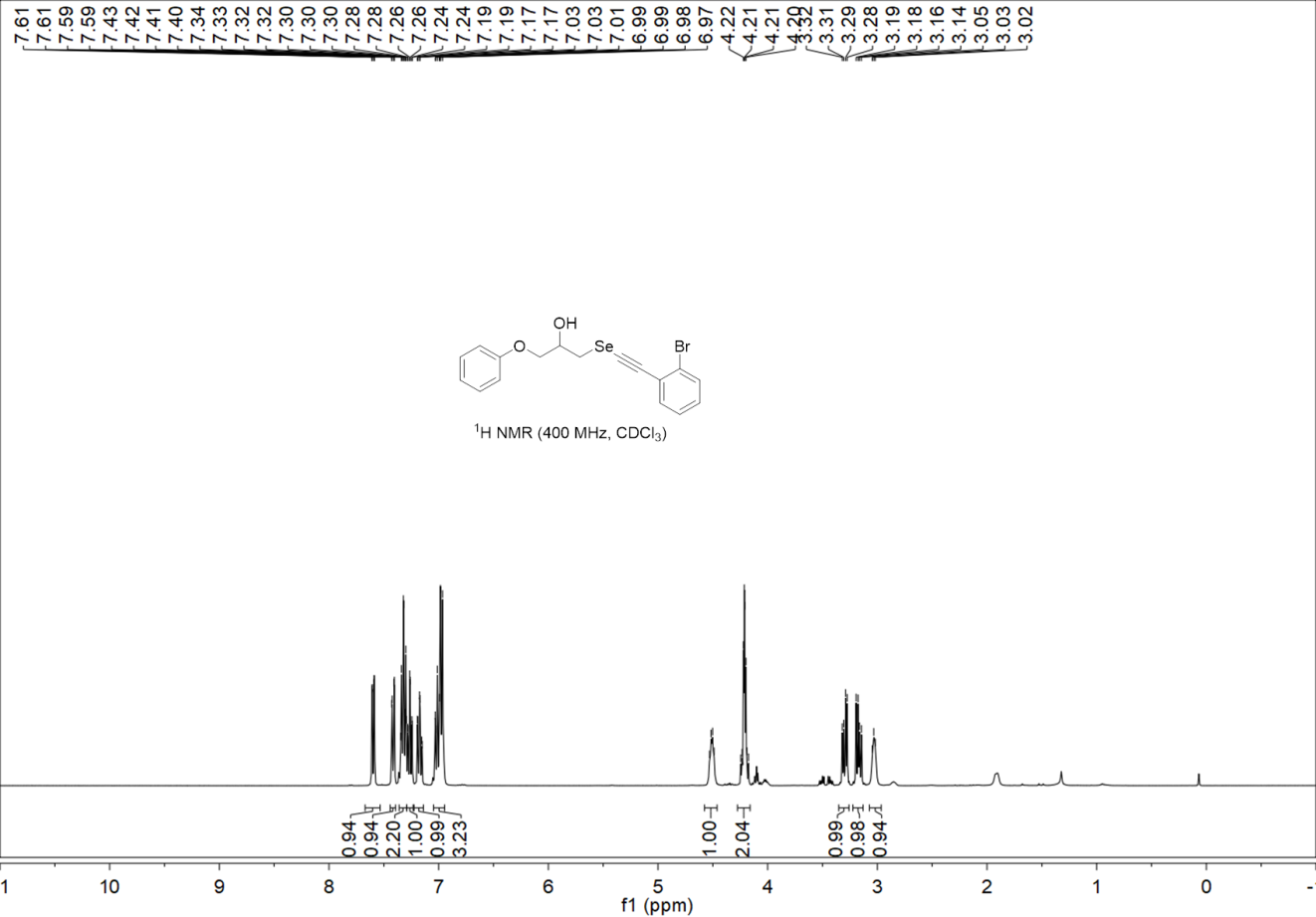


**Fig. S7** ^1^H NMR spectrum of compound **3h-2**


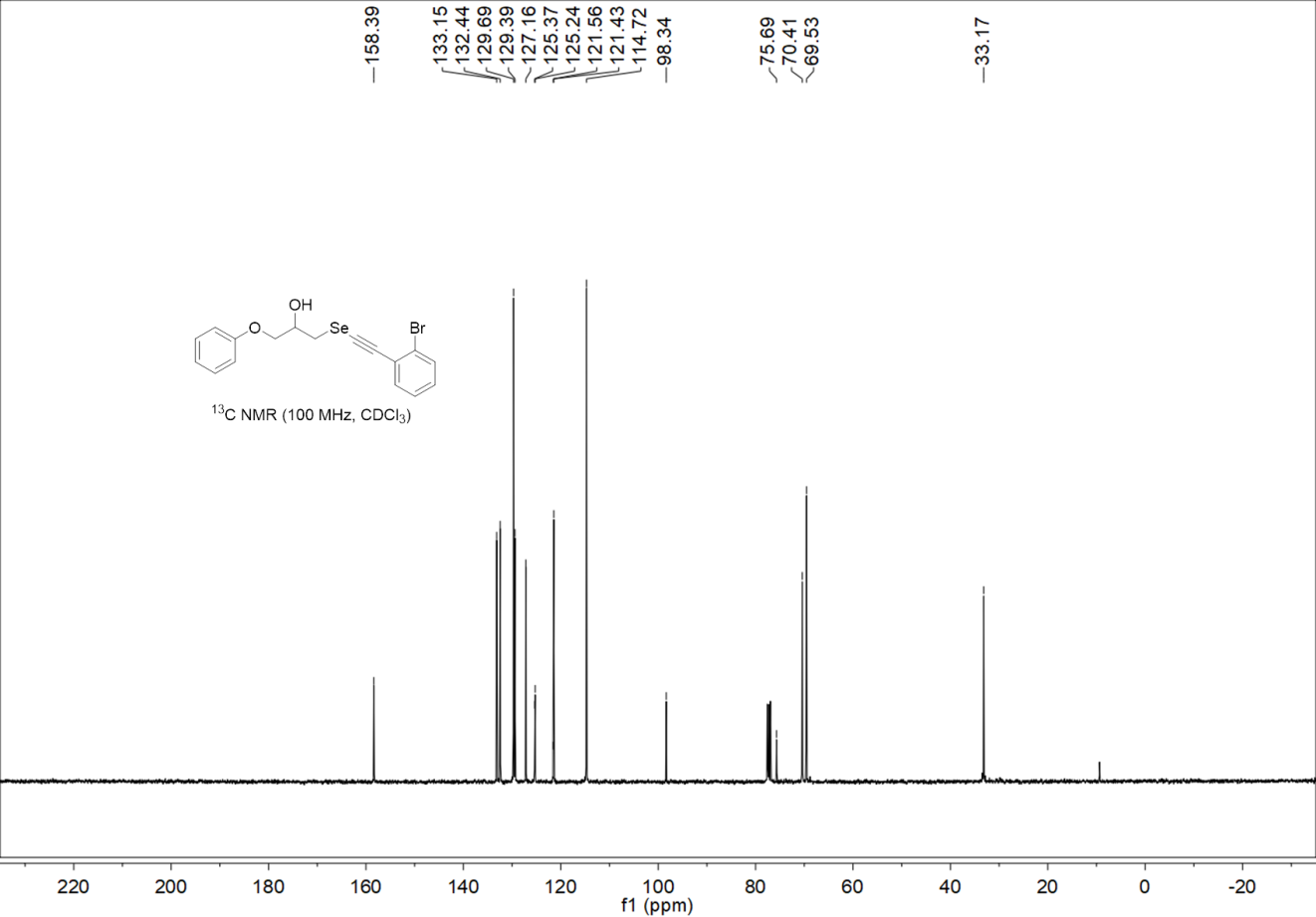


**Fig. S8** ^13^C NMR spectrum of compound **3h-2**


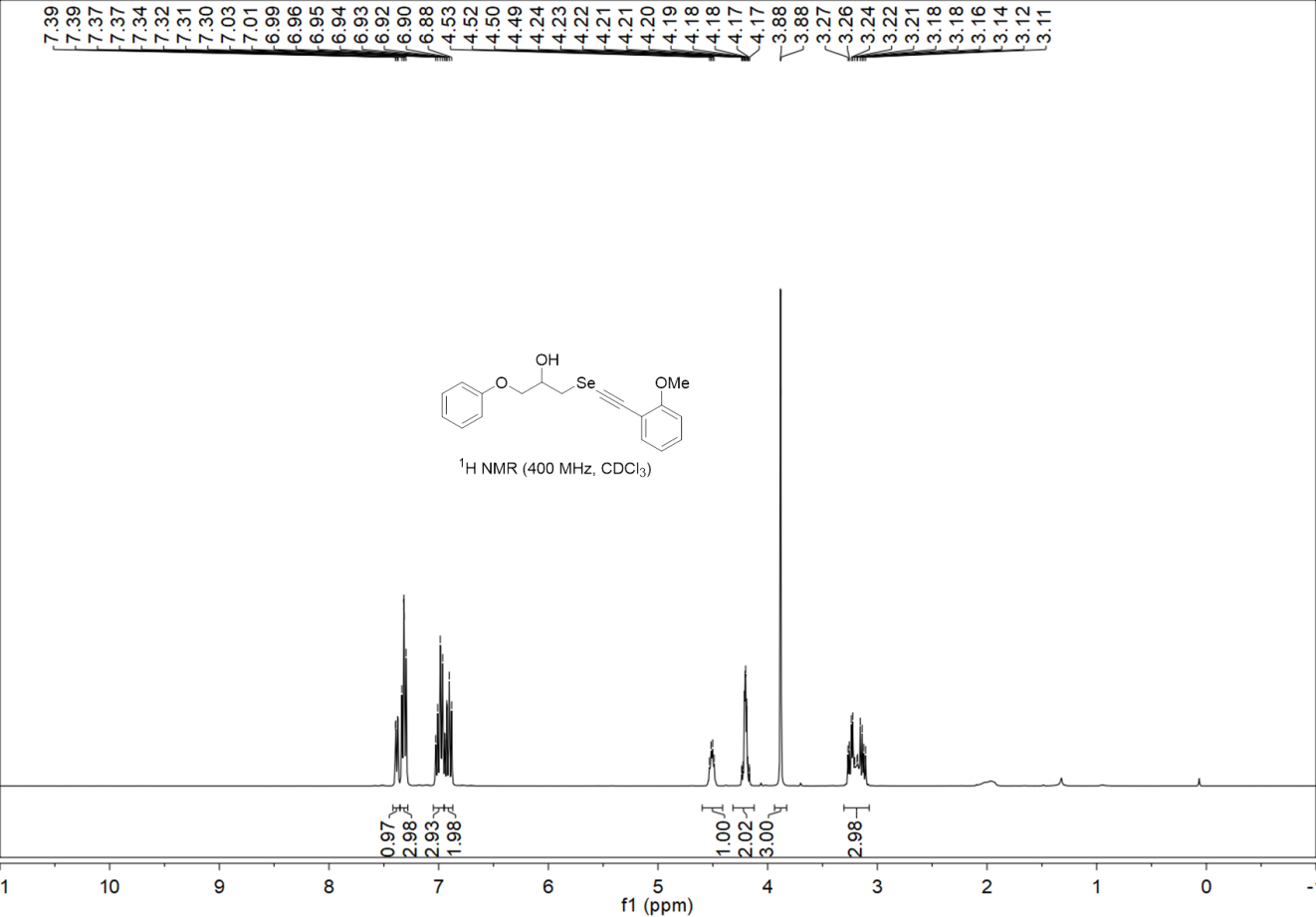


**Fig. S9** ^1^H NMR spectrum of compound **3h-3**


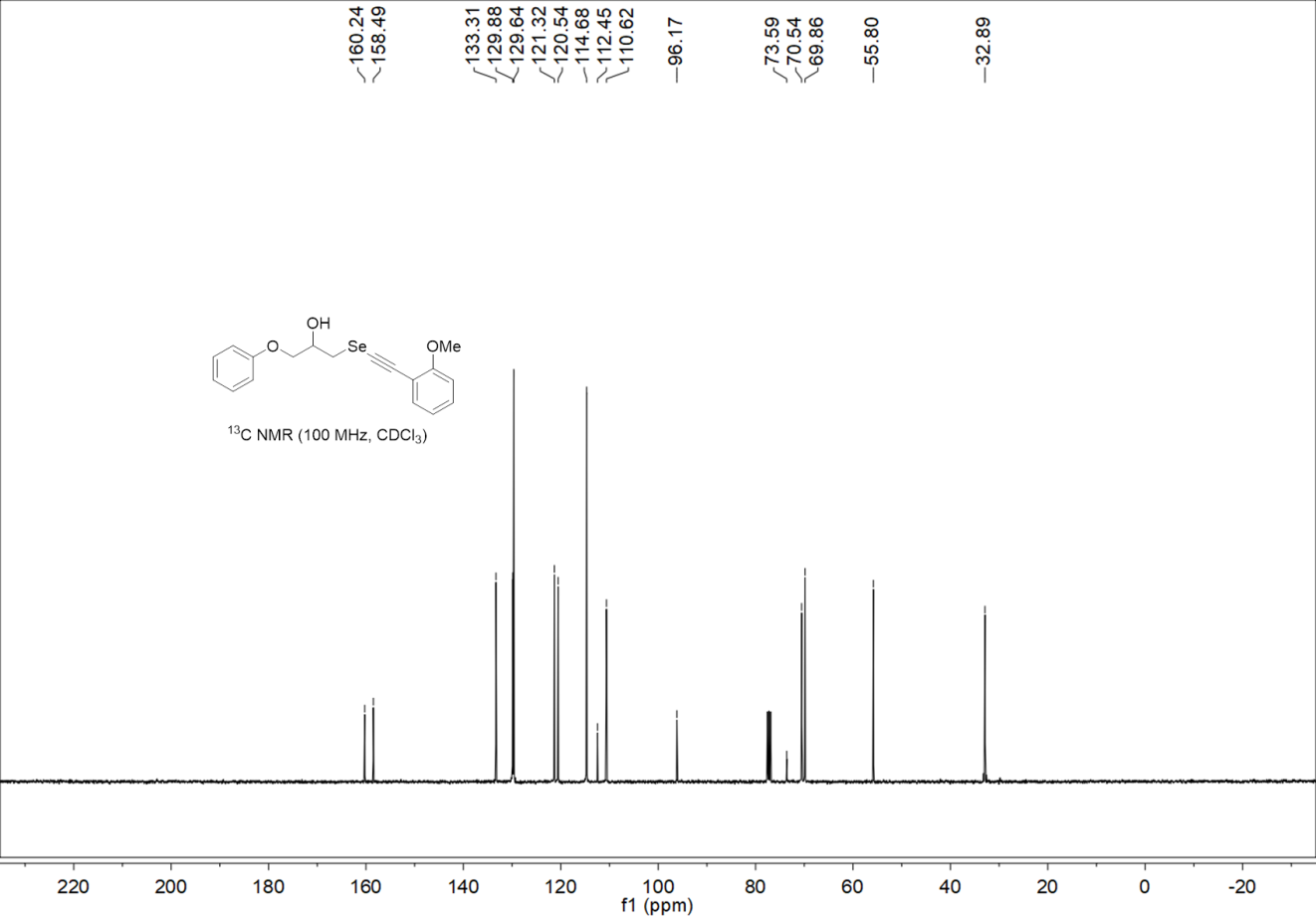


**Fig. S10** ^13^C NMR spectrum of compound **3h-3**
